# Plasma metabolomic signatures of air pollution and rheumatoid arthritis risk

**DOI:** 10.3389/fpubh.2026.1901951

**Published:** 2026-08-12

**Authors:** Yangchang Zhang, Tian Liang, Yang Pu, Linpeng Zhou, Lingli Ding, Chengyin Li, Xun Lei, Bin Wu

**Affiliations:** 1 Department of Rheumatology and Immunology, The First Affiliated Hospital of Chongqing University of Chinese Medicine, Chongqing, China; 2 School of Public Health, Capital Medical University, Beijing, China; 3 School of General Education, Chongqing University of Chinese Medicine, Chongqing, China; 4 School of Public Health, Chongqing Medical University, Chongqing, China

**Keywords:** air pollution, cohort, mediation, metabolism, rheumatoid arthritis

## Abstract

**Introduction:**

Ambient air pollutants have been increasingly implicated in immune-mediated disorders, yet the metabolic perturbations through which long-term exposure may contribute to rheumatoid arthritis (RA) remain insufficiently characterized.

**Methods:**

We used data from 403,332 RA-free participants in the UK Biobank cohort to investigate the associations among long-term ambient air pollution exposure, plasma metabolic signatures, and incident RA risk. Participants were followed for a median of 14.01 years. Residential annual average concentrations of PM_2.5_, PM_10_, NO_2_, and NO_x_ were linked to participants' addresses, and a composite air pollution score (APS) was calculated to reflect overall exposure burden. Plasma metabolomics data were used to characterize metabolic perturbations related to each pollutant. A machine learning model was applied to identify air pollutant-associated metabolites and construct corresponding metabolic signatures. We then examined the relationships of air pollutants and derived metabolic signatures with RA risk using Cox proportional hazards models. Generalized propensity score analyses were performed to assess whether the findings persisted after covariate balancing, and mediation analyses were conducted to estimate the contribution of metabolic signatures to the relationship of air pollutants and RA.

**Results:**

Each standard deviation increase in the ${\text{PM}}_{2.5^{-}}$, ${\text{NO}}_{2^{-}}$, ${\text{NO}}_{{\text{x}}^{-}}$, and APS-related metabolic signatures was associated with elevated RA risk, with hazard ratios of 1.208 (95% CI: 1.180, 1.236), 1.141 (95% CI: 1.109, 1.173), 1.174 (95% CI: 1.148, 1.201), and 1.184 (95% CI: 1.156, 1.212), respectively. Similar estimates were observed in generalized propensity score analyses. Metabolic signatures accounted for 15.7% to 21.1% of the associations between air pollution exposure and RA risk.

**Conclusion:**

These findings suggest that long-term air pollution exposure is associated with incident RA risk and that systemic metabolic perturbations may represent potential biological pathways underlying this association.

## 1 Introduction

Rheumatoid arthritis (RA) is an autoimmune disease with a substantial clinical and public health burden. Emerging evidence suggests that the global burden of RA has risen steadily over recent decades (1, 2). In 2021, an estimated 17.9 million individuals were living with RA, representing a 125% increase compared with 1990 (2). RA prevalence varies markedly by geographic region and urban–rural setting, and is generally higher in industrialized countries (3, 4). Therefore, RA imposes a substantial public health burden through chronic disability and high healthcare costs. Genetic evidence also indicated the associations of PM_2.5_ (particulate matter) exposure with inflammation, such as asthma and chronic obstructive pulmonary disease (5).

Growing evidence suggests that long-term exposure to ambient air pollution may be associated with the development and progression of RA (6–13). The investigation of air pollution exposure and systemic metabolism is a nascent and novel area. A systematic review and meta-analysis by Di et al. summarized available epidemiological evidence on long-term outdoor air pollution and RA risk and suggested a possible positive association, although heterogeneity and inconsistent findings across studies remained (11). Subsequent large population-based studies have provided further evidence. For example, cohort studies have linked sustained exposure to fine particulate matter and nitrogen-related air pollutants with incident RA (6–8), whereas some earlier studies, including the Nurses' Health Study and the Swedish EIRA study, reported inconsistent associations (12, 13). A large Canadian cohort study by Zhao et al. reported associations between long-term exposure to PM_2.5_ components and RA risk, suggesting that different particulate constituents may have distinct immunological relevance (14). In addition, a Mendelian randomization study by Hu et al. provided genetic-instrument evidence supporting potential relationships between air pollution and autoimmune diseases, although such findings require careful interpretation given the assumptions of Mendelian randomization (15). Multi-omics evidence by Zhao et al. suggested that PM_10_ exposure may contribute to immune-cell dysfunction and RA progression, providing mechanistic support for the involvement of pollution-related immune perturbation (16).

A short-term exposure study in humans indicate that traffic-related air pollution can induce acute alterations in the serum metabolome, affecting pathways related to inflammation and oxidative stress (17). This suggests that chronic exposure may induce persistent metabolic alterations that contribute to disease pathogenesis. Studies have also identified distinct metabolites associated with RA risk. For example, a longitudinal study identified 81 metabolites associated with RA risk, which partially mediated the protective effect of a healthy lifestyle (18). Another study identified the differential metabolites between RA and healthy adults, and revealed causal associations using Mendelian randomization and genetic correlation. This study found that the metabolic risk score comprising 17 metabolites underpinned RA risk, and high genetic risk further amplified this association (19). However, large-scale prospective evidence linking air pollution-related metabolic signatures to future RA risk is still limited.

We investigated the mediating effects of circulating metabolomic profiles in the association between air pollution exposure and RA risk using a large prospective design from UK Biobank.

## 2 Method

### 2.1 . Study population

UK Biobank is a prospective cohort comprising more than 500,000 adults aged 40–69 years who were recruited between 2006 and 2010 in the UK. Baseline assessments included questionnaire data on sociodemographic factors, lifestyle, and medical history, together with physical examinations and biological sample collection. Participants' health outcomes have been followed through linkage to routinely collected national health records (20). All participants gave written informed consent, and ethical approval was provided by the North West Multi-center Research Ethics Committee (number: 21/NW/0157).

The baseline cohort comprised 502,007 participants. We sequentially excluded individuals who had missing data for the 251 metabolites or whose biospecimens did not meet quality-control criteria, including samples flagged for high lactate, high pyruvate, low glucose, or low protein (*n* = 45,944). We further removed participants with missing or invalid exposure estimates for PM_2.5_, PM_10_, NO_2_, or NO_x_ (*n* = 42,125), as well as those with prevalent RA at baseline or RA diagnosed during the first 5 years of follow-up to minimize potential reverse causation and the influence of preclinical or undiagnosed RA at baseline (*n* = 10,606) (21). Finally, 403,332 participants were included in further analyses (Supplementary Figure S1).

### 2.2 . Metabolic profiles

Circulating metabolites were quantified in EDTA plasma (aliquot 3) using the high-throughput nuclear magnetic resonance (NMR) platform developed by Nightingale Health (Helsinki, Finland) (22). The NMR metabolomics platform provides 251 metabolic traits for each sample, comprising 170 directly quantified concentrations and 81 derived ratios. These measures cover a broad range of biological pathways, including lipoprotein subclasses, inflammatory markers, fatty acids, and amino acids, and are mainly reported as absolute concentrations in mmol/L or as ratios. Quality control procedures were implemented at multiple stages of sample handling and data acquisition. These included automated assessment of spectral quality, continuous evaluation of intra- and inter-spectrometer performance, and the use of two internal controls together with two blinded duplicate samples on every 96-well plate. Across sequential analytical batches, the platform met predefined reproducibility criteria, with many biomarkers showing coefficients of variation below 5%. Sample- and biomarker-level QC indicators were further applied to detect possible degradation, contamination, dilution effects, or unreliable quantification. Missing metabolite concentrations were replaced with half the limit of detection, consistent with previous studies (23, 24). To ensure comparability across biomarkers measured on different scales, all metabolic traits were standardized to Z scores within the analytic population, with a mean of 0 and a standard deviation of 1. Detailed information and procedures have been reported elsewhere (25).

### 2.3 . Air pollutants

Baseline exposure to ambient air pollution was assessed using annual mean concentrations of NO_x_ (nitrogen oxides), NO_2_ (nitrogen dioxide), PM_10_ (inhalable particulate matter), and PM_2.5_. These estimates were generated from land-use regression models developed within the ESCAPE project. The 2010 annual average concentrations were selected as the main baseline exposure indicators (6, 26). To represent the overall exposure burden from multiple correlated pollutants, we developed a weighted air pollution score (APS) based on PM_2.5_, NO_2_, and NO_x_. For each pollutant, the weight was defined using the regression coefficient from the corresponding fully adjusted single-pollutant model. The APS was calculated as follows: APS = (β_PM2.5_×PM_2.5_+β_NO2_×NO_2_+β_NOx_×NO_x_) × (3/sum of the β coefficients) (23).

For the analysis of long-term exposure, we further compiled a time-varying exposure dataset using annual mean concentrations of PM_2.5_, PM_10_, NO_2_, and NO_x_ from 2003 to 2023 obtained from DEFRA. These data were produced by a UK-wide air dispersion model with a spatial resolution of 1 × 1 km. The model integrated source-specific emissions from the National Atmospheric Emissions Inventory and information on secondary inorganic aerosols. Annual exposures were assigned according to individual residential address histories by matching each address to the nearest 1 × 1 km modeled grid point.

### 2.4 . Outcome ascertainment

The follow-up period started at baseline and ended at the first diagnosis of rheumatoid arthritis, death, loss to follow-up, or March 31, 2023, whichever occurred earliest. Incident rheumatoid arthritis cases were identified through linkage to hospital inpatient records using ICD-10 codes M05 and M06.

### 2.5 . Assessment of covariates

Covariates were prespecified according to prior evidence and knowledge, with further guidance from a directed acyclic graph (DAG; Supplementary Figure S2) (27, 28). The adjusted variables covered demographic and socioeconomic factors, lifestyle behaviors, adiposity, renal function, lipid profiles, and baseline comorbidities. Specifically, these included age, sex, ethnicity, educational attainment, employment status, household income, smoking status, alcohol intake, healthy dietary pattern, physical activity, body mass index (BMI), estimated glomerular filtration rate (eGFR), low-density lipoprotein cholesterol, natural log-transformed triglycerides, and prevalent diabetes, hypertension, and cardiovascular disease.

Ethnicity was classified as White or non-White. Education was grouped as college or above, high school or equivalent, and less than high school; employment status as employed, retired, or unemployed; and household income as < £31,000 or ≥ £31,000. Smoking and alcohol intake were categorized as never, previous, or current. Physical activity was calculated as total MET-minutes per week and dichotomized into low and high levels using the median. Diet quality was assessed using a five-point score based on higher intake of vegetables, fruits, and fish and lower intake of red and processed meat, with a score ≥4 indicating a healthy dietary pattern. BMI was classified as < 25.0, 25.0–30.0, >30.0 kg/m^2^. Baseline comorbidities were treated as yes/no variables, including cardiovascular disease, diabetes, and hypertension. Missing values were replaced by the median for continuous covariates and the mode for categorical covariates.

### 2.6 . Statistical analysis

All metabolic biomarkers were standardized to Z scores before analysis. To identify air pollution-related metabolic signatures, we applied least absolute shrinkage and selection operator (LASSO) regression using the glmnet package in R. For each pollutant and the air pollution score (APS), standardized metabolites were entered as candidate predictors. The tuning parameter was selected by 10-fold cross-validation, and λ1se was used to obtain a more parsimonious model. Metabolites with non-zero coefficients at λ1se were retained, and pollutant-related metabolic signatures were calculated as weighted sums of the selected metabolites using their LASSO-derived coefficients.

Cox proportional hazards models were used to examine the associations of air pollutants and pollutant-related metabolic signatures with incident rheumatoid arthritis (RA). Hazard ratios (HRs) and 95% confidence intervals (CIs) were reported. The fully adjusted model included age, sex, ethnicity, educational attainment, employment status, household income, body mass index, physical activity, smoking status, alcohol intake, healthy dietary pattern, low-density lipoprotein cholesterol, natural log-transformed triglycerides, diabetes, hypertension, cardiovascular disease, and estimated glomerular filtration rate. The proportional hazards assumption was assessed using Schoenfeld residuals.

We further evaluated exposure–response relationships using restricted cubic splines, mixture effects using quantile-based g-computation, and covariate balance using generalized propensity score (GPS) weighting. Exploratory mediation analyses were conducted to assess whether baseline metabolic signatures partly explained the associations between long-term air pollution exposure and subsequent RA risk, assuming the temporal sequence of long-term exposure, baseline metabolomic profile, and incident RA during follow-up. Additional sensitivity analyses were performed to test the robustness of the findings. Detailed statistical procedures are provided in the Supplementary Methods. All analyses were performed using R version 4.4.1, and two-sided *P* values < 0.05 were considered statistically significant.

## 3 Results

### 3.1 . Population characteristics

The final analytic sample included 403,332 participants, with an average baseline age of 56.47 years. Over a median follow-up period of 14.01 years, 4,973 participants developed RA, corresponding to a cumulative incidence of 1.23%. Given the large sample size, standardized mean differences (SMDs) were used to assess the magnitude of baseline differences. Participants with incident RA were older and were more frequently women, had lower educational attainment and household income, were more likely to be retired, and had a higher prevalence of obesity, hypertension, diabetes, and cardiovascular disease. Although above differences were statistically significant (all *P* < 0.05), their magnitudes varied according to SMDs, with relatively larger differences observed for age, employment status, educational attainment, sex, BMI category, and household income (Table 1). In 2010, the mean concentrations of PM_2.5_, PM_10_, NO_2_, and NO_x_ were 9.97, 16.21, 26.48, and 43.50 μg/m^3^, respectively, with corresponding standard deviations of 1.02, 1.87, 7.22, and 13.95 μg/m^3^ (Supplementary Table S2).

**Table 1:** Demographic characteristics across rheumatoid arthritis status in the study (*n* = 403,332).

Variables	Health participants	Incident RA	*P*	SMD
	*N* = 398,359	*N* = 4,973		
Age (year), mean (SD)	56.43 (8.09)	59.54 (7.14)	<0.001	0.407
Sex, *n* (%)				0.237
Male	180,258 (45.3)	1,678 (33.7)	<0.001	
Female	218,101 (54.7)	3,295 (66.3)		
Ethnicity, *n* (%)				0.024
White	374,560 (94.0)	4,647 (93.4)	0.092	
Non-white	23,799 (6.0)	326 (6.6)		
Education, *n* (%)				0.316
College or above	179,854 (45.1)	1,633 (32.8)	<0.001	
High school or equivalent	153,789 (38.6)	1,971 (39.6)		
Less than high school	64,716 (16.2)	1,369 (27.5)		
Employment status, *n* (%)				0.374
Employed	236,510 (59.4)	2,038 (41.0)	<0.001	
Retired	130,222 (32.7)	2,359 (47.4)		
Unemployed	31,627 (7.9)	576 (11.6)		
Household income (£), *n* (%)				0.202
<31,000	160,243 (40.2)	2,498 (50.2)	<0.001	
>= 31,000	238,116 (59.8)	2,475 (49.8)		
Physical activity, *n* (%)				0.086
Low	248,783 (62.5)	3,310 (66.6)	<0.001	
High	149,576 (37.5)	1,663 (33.4)		
Smoking status, *n* (%)				0.177
Never	222,275 (55.8)	2,343 (47.1)	<0.001	
Previous	136,841 (34.4)	2,000 (40.2)		
Current	39,243 (9.9)	630 (12.7)		
Alcohol intake, *n* (%)				0.126
Never	17,378 (4.4)	324 (6.5)	<0.001	
Previous	13,578 (3.4)	246 (4.9)		
Current	367,403 (92.2)	4,403 (88.5)		
Healthy diet pattern, *n* (%)				0.022
No	267,402 (67.1)	3,287 (66.1)	0.129	
Yes	130,957 (32.9)	1,686 (33.9)		
Body mass index, n (%)				0.235
<25	133,125 (33.4)	1,269 (25.5)	<0.001	
25-30	172,412 (43.3)	2,070 (41.6)		
> 30	92,822 (23.3)	1,634 (32.9)		
Cardiovascular diseases, *n* (%)				0.142
No	376,836 (94.6)	4,522 (90.9)	<0.001	
Yes	21,523 (5.4)	451 (9.1)		
Diabetes, *n* (%)				0.118
No	376,743 (94.6)	4,554 (91.6)	<0.001	
Yes	21,616 (5.4)	419 (8.4)		
Hypertension, *n* (%)				0.174
No	197,475 (49.6)	2,035 (40.9)	<0.001	
Yes	200,884 (50.4)	2,938 (59.1)		
eGFR (ml/min/1.73m^3^), mean (SD)	90.83 (12.52)	88.62 (13.03)	<0.001	0.173
LDL (mmol/L), mean (SD)	3.55 (0.83)	3.52 (0.85)	0.021	0.032
TG (mmol/L), median (Q1, Q3)	1.48 [1.06, 2.03]	1.48 [1.14,2.15]	<0.001	0.109

eGFR, estimated glomerular filtration rate; LDL, low-density lipoprotein; TG, triglycerides; SMD: Standardized mean difference. An absolute SMD <0.10 was considered to indicate a negligible difference between groups. The SMD for triglycerides was calculated using natural log-transformed values.

### 3.2 . Identification of air pollution-related metabolic signatures

**Figure 1:**
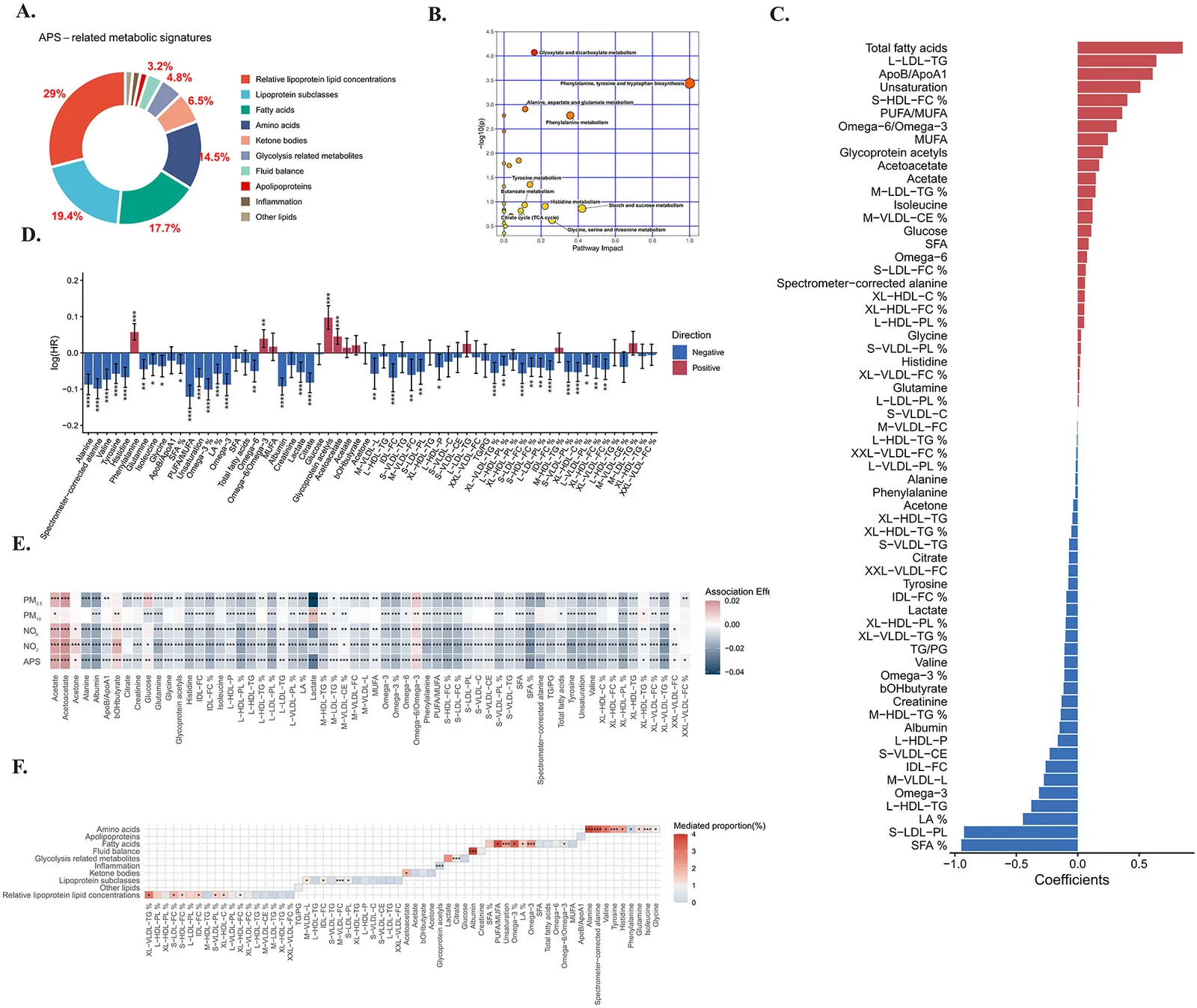
Associations of metabolites comprising the air pollution score-related metabolic signature with rheumatoid arthritis. **(A)** Constituent metabolites of the APS-related metabolic signature. **(B)** Pathway analysis of significant mediated metabolites consisted of APS-related metabolic signature. The x-axis indicates pathway impact, whereas the y-axis reflects the significance of each pathway. Larger and darker bubbles indicate pathways with greater impact and higher significance. **(C)** Metabolite coefficients from LASSO regression in the APS-related metabolic signature. **(D)** The effects of metabolites comprising the APS-related metabolic signature on rheumatoid arthritis using Cox regression model. Model adjusted for age, sex, ethnicity, education levels, employment status, household income, body mass index, physical activity, smoking status, alcohol intake, healthy diet pattern, cardiovascular diseases history, diabetes history, hypertension history, estimated glomerular filtration rate, low-density lipoprotein, natural logarithm of triglycerides. The effect size is presented as log (HR), with 95% confidence intervals shown as error bars. Red bars indicate an increased risk, whereas blue bars indicate a decreased risk. Statistical significance is denoted by asterisks (^*^P < 0.05, ^**^P < 0.01, ^***^P < 0.001). **(E)** Associations of individual air pollutants and the APS with metabolites comprising the APS-related metabolic signature, estimated using multivariable linear regression. The model adjustments are consistent with model in Plot D. The effect size is presented as beta and the significance is labeled by asterisks (^*^P < 0.05, ^**^P < 0.01, ^***^P < 0.001). **(F)** Mediating effects of the identified metabolites on the association between APS and rheumatoid arthritis. The mediated analysis is conducted and adjusted for determinants in Plot D. The mediating proportion was calculated by dividing the indirect effects by the total effects. Significance is indicated by asterisks (^*^P < 0.05, ^**^P < 0.01, ^***^P < 0.001).

Air pollution indicators (i.e., PM_2.5_, PM_10_, NO_2_, NO_x_, and APS) were significantly associated with a broad range of metabolites. LASSO regression identified 48, 25, 89, 67, and 62 metabolites for construct the metabolic signatures of PM_2.5_, PM_10_, NO_2_, NO_x_, and APS, respectively (Supplementary Figures S3–S6 and Figure 1C). These metabolites spanned multiple metabolomic classes (Supplementary Figure S7). The APS-related metabolic signature was predominantly characterized by lipoprotein lipid measures, lipoprotein subclasses, fatty acids, and amino acids (Figure 1A). Pathway enrichment analysis indicated that these metabolites were predominantly involved in glyoxylate and dicarboxylate metabolism; phenylalanine, tyrosine and tryptophan biosynthesis; phenylalanine metabolism; and alanine, aspartate and glutamate metabolism (Figure 1B and Supplementary Table S3). Within the APS-related signature, total fatty acids and triglycerides in large LDL (L-LDL-TG) had the largest positive coefficients, whereas phospholipids in small LDL (S-LDL-PL) and the ratio of saturated fatty acids to total fatty acids (SFA%) had the largest negative coefficients (Figure 1C).

We investigated the associations of the selected metabolites with RA risk (Figure 1D and Supplementary Figures S3–S6). For APS, most metabolites were inversely associated with the RA, whereas only a limited number showed positive associations (Figure 1D). Among the positively associated metabolites, glycoprotein acetyls, phenylalanine, acetoacetate, and omega-6 exhibited relatively larger effect estimates. In contrast, a broader range of metabolites, particularly several lipid-related traits and lipoprotein subclass components, showed negative associations. Similar patterns were observed for PM_2.5_, PM_10_, NO_2_, and NO_x_ (Supplementary Figures S3–S6). We also found the selected metabolites were significantly linked with multiple air pollutants or APS (Figure 1E and Supplementary Figures S3–S6). We additionally evaluated whether individual metabolites mediated the association with RA. For APS, higher mediated proportions were mainly observed for amino acids (e.g., alanine, valine, tyrosine and isoleucine), fatty acids (e.g., the ratio of polyunsaturated fatty acids to monounsaturated fatty acids [PUFA/MUFA], degree of unsaturation [unsaturation], the ratio of omega-3 fatty acids to total fatty acids [Omega-3 %], the ratio of linoleic acid to total fatty acids [LA %], and Omega-3), and fluid balance (i.e., albumin) (Figure 1F).

### 3.3 . Associations of air pollutants and metabolic signatures with the risk of RA

After multivariable adjustment, higher air pollution exposure was associated with an increased risk of incident RA. Each SD increment in PM_2.5_, PM_10_, NO_2_, NO_x_, and APS was associated with 4.6%, 2.5%, 3.9%, 4.3%, and 4.5% higher RA risk, respectively (Table 2). When exposures were categorized into tertiles, participants in the highest tertile had higher RA risk than those in the lowest tertile, with fully adjusted HRs of 1.137 (95% CI: 1.060, 1.219) for PM_2.5_, 1.110 (95% CI: 1.036, 1.189) for PM_10_, 1.110 (95% CI: 1.033, 1.192) for NO_2_, 1.091 (95% CI: 1.016, 1.170) for NO_x_, and 1.120 (95% CI: 1.043, 1.201) for APS (Table 2).

**Table 2:** Association of air pollutants and air pollution score with rheumatoid arthritis risk among UK adults.

Air pollutants	Model 1		Model 2		Model 3	
	HR (95% CI)	*P* value	HR (95% CI)	*P* value	HR (95% CI)	*P* value
PM_2.5_						
Tertile 1	Ref		Ref		Ref	
Tertile 2	1.148 (1.071–1.230)	< 0.001	1.113 (1.038–1.193)	0.003	1.086 (1.013–1.165)	0.020
Tertile 3	1.259 (1.175–1.349)	< 0.001	1.200 (1.119–1.286)	< 0.001	1.137 (1.060–1.219)	< 0.001
P for trend		< 0.001		< 0.001		< 0.001
Per SD increment	1.096 (1.067–1.127)	< 0.001	1.072 (1.043–1.103)	< 0.001	1.046 (1.017–1.076)	0.002
PM_10_						
Tertile 1	Ref		Ref		Ref	
Tertile 2	1.075 (1.003–1.152)	0.041	1.057 (0.986–1.132)	0.119	1.036 (0.966–1.110)	0.323
Tertile 3	1.158 (1.081–1.240)	< 0.001	1.144 (1.068–1.225)	< 0.001	1.110 (1.036–1.189)	0.003
P for trend		< 0.001		< 0.001		0.002
Per SD increment	1.047 (1.019–1.076)	0.001	1.038 (1.01–1.067)	0.008	1.025 (0.997–1.054)	0.081
NO_2_						
Tertile 1	Ref		Ref		Ref	
Tertile 2	1.210 (1.129–1.296)	< 0.001	1.158 (1.081–1.241)	< 0.001	1.123 (1.048–1.204)	0.001
Tertile 3	1.211 (1.128–1.299)	< 0.001	1.174 (1.093–1.260)	< 0.001	1.110 (1.033–1.192)	0.004
P for trend		< 0.001		< 0.001		0.005
Per SD increment	1.079 (1.049–1.110)	< 0.001	1.065 (1.035–1.096)	< 0.001	1.039 (1.009–1.069)	0.010
NO_x_						
Tertile 1	Ref		Ref		Ref	
Tertile 2	1.139 (1.063–1.221)	< 0.001	1.098 (1.024–1.176)	0.008	1.065 (0.994–1.141)	0.076
Tertile 3	1.208 (1.127–1.294)	< 0.001	1.156 (1.078–1.240)	< 0.001	1.091 (1.016–1.170)	0.016
P for trend		< 0.001		< 0.001		0.017
Per SD increment	1.088 (1.059–1.118)	< 0.001	1.069 (1.040–1.099)	< 0.001	1.043 (1.014–1.072)	0.003
APS						
Tertile 1	Ref		Ref		Ref	
Tertile 2	1.148 (1.071–1.231)	< 0.001	1.107 (1.032–1.187)	0.004	1.074 (1.002–1.152)	0.043
Tertile 3	1.240 (1.156–1.329)	< 0.001	1.187 (1.107–1.273)	< 0.001	1.120 (1.043–1.201)	0.002
P for trend		< 0.001		< 0.001		0.002
Per SD increment	1.093 (1.063–1.124)	< 0.001	1.073 (1.043–1.103)	< 0.001	1.045 (1.016–1.075)	0.002

PM_2.5_, particulate matter with an aerodynamic diameter ≤ 2.5 μm; PM_10_, particulate matter with an aerodynamic diameter ≤ 10 μm; NO_2_, nitrogen dioxide; NO_x_, nitrogen oxides; APS: Air pollution score; HR, hazard ratio; CI, confidence interval. Results are presented as HRs and 95% CIs per SD increase in air pollutants. Model 1: adjusted age, sex and ethnicity. Model 2: further adjusted for education levels, employment status, and household income. Model 3: further adjusted for body mass index, physical activity, smoking status, alcohol intake, healthy diet pattern, cardiovascular diseases history, diabetes history, hypertension history, estimated glomerular filtration rate, low-density lipoprotein, natural logarithm of triglycerides.

In mixture analyses using quantile g-computation, a one-quartile increase in the pollutant mixture was associated with increased RA risk, with HRs of 1.054 (95% CI: 1.022, 1.086) for the PM_2.5_-PM_10_-NO_2_ mixture and 1.056 (95% CI: 1.024, 1.088) for the PM_2.5_-PM_10_-NO_x_ mixture. The joint effects of metabolic signature mixtures ranged from 1.114 to 1.216 (Supplementary Table S4). Stratified analyses showed stronger associations of PM_10_, NO_2_, and NO_x_ with RA among women than among men (*P* for interaction < 0.05; Supplementary Figure S8). Restricted cubic spline analyses showed significant nonlinear associations of PM_2.5_ and NO_x_ with RA risk (*P* for nonlinearity = 0.048 and 0.046, respectively). The exposure–response curves generally increased at lower exposure levels and then tended to plateau at higher concentrations (Supplementary Figure S9).

**Figure 2:**
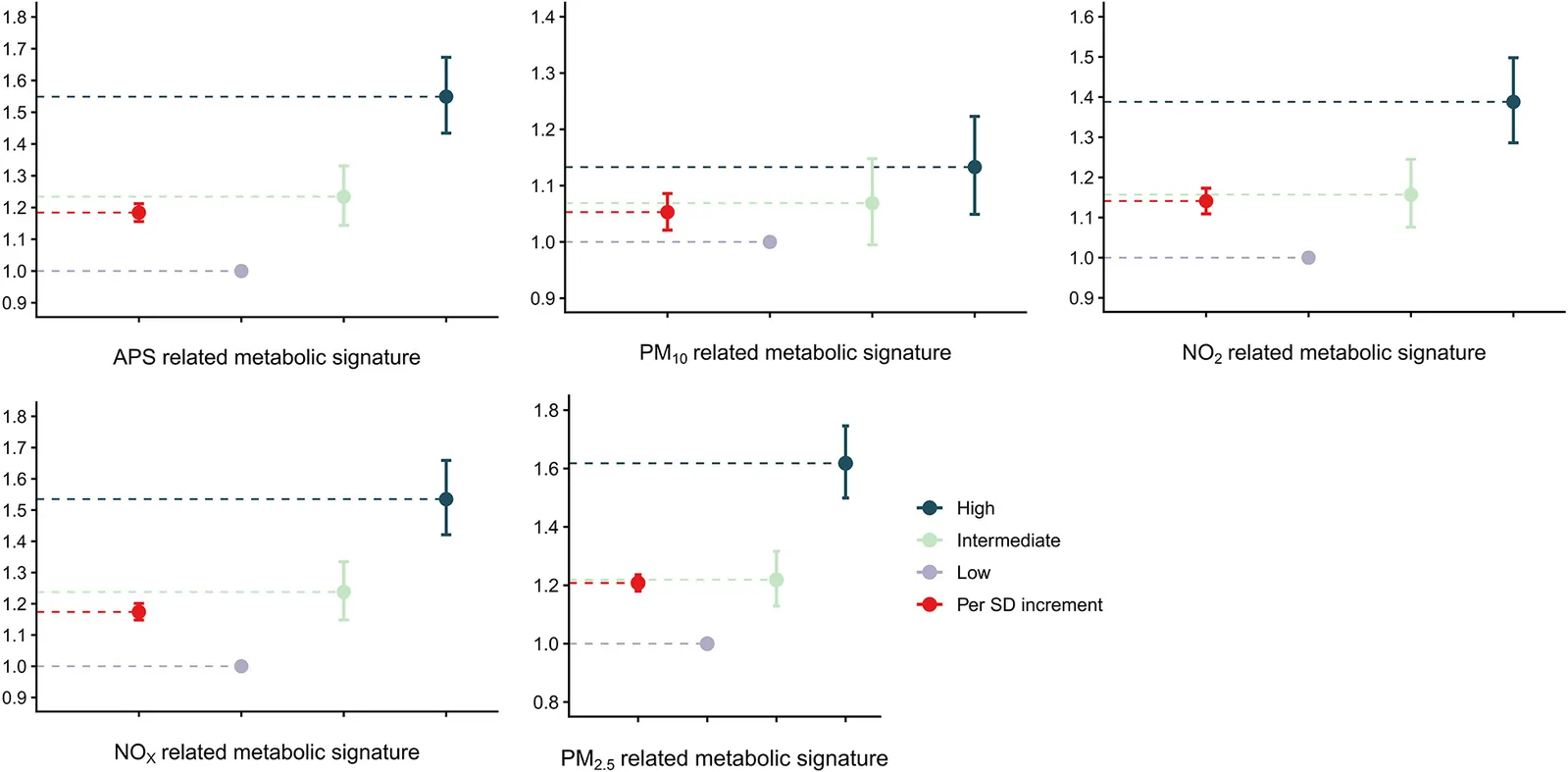
Associations of air pollution related metabolic signatures with rheumatoid arthritis. Model adjusted for age, sex, ethnicity, education levels, employment status, household income, body mass index, physical activity, smoking status, alcohol intake, healthy diet pattern, cardiovascular diseases history, diabetes history, hypertension history, estimated glomerular filtration rate, low-density lipoprotein, natural logarithm of triglycerides.

Pollutant-related metabolic signatures were consistently associated with a higher RA risk. Per SD increment, the fully adjusted HRs were 1.208 (95% CI: 1.180, 1.236) for the PM_2.5_- related signature, 1.053 (95% CI: 1.021, 1.086) for the PM_10_- related signature, 1.141 (95% CI: 1.109, 1.173) for the NO_2_- related signature, 1.174 (95% CI: 1.148, 1.201) for the NO_x_- related signature, and 1.184 (95% CI: 1.156, 1.212) for the APS-related signature (Figure 2). Participants in the highest tertile of these signatures also had higher RA risk than those in the lowest tertile, with HRs ranging from 1.133 for the PM_10_- related signature to 1.618 for the PM_2.5_- related signature (Figure 2).

### 3.4 . Weighted analyses of air pollution, metabolic signatures, and RA risk

Weighted Cox regression yielded results broadly comparable to those observed in the main analyses (Table 3). After applying GPS weighting, air pollutants remained positively associated with RA risk. The estimated HRs were 1.056 (95% CI: 1.028, 1.085) for PM_2.5_, 1.029 (95% CI: 1.002, 1.057) for PM_10_, 1.045 (95% CI: 1.017, 1.074) for NO_2_, and 1.051 (95% CI: 1.023, 1.080) for NO_x_. Similar patterns were observed for pollutant-related metabolic signatures, with HRs of 1.203 (95% CI: 1.178, 1.230), 1.049 (95% CI: 1.020, 1.079), 1.133 (95% CI: 1.105, 1.163), and 1.168 (95% CI: 1.145, 1.192) for the ${\text{PM}}_{2.5^{-}}$, ${\text{PM}}_{10^{-}}$, ${\text{NO}}_{2^{-}}$, and ${\text{NO}}_{{\text{x}}^{-}}$ related signatures, respectively (Table 3).

**Table 3:** Weighted associations of air pollution and related metabolic signatures with rheumatoid arthritis.

Exposure	HR (95% CI)	*P* value	Exposure	HR (95% CI)	*P* value
Air pollution			Metabolic signature		
PM_2.5_	1.056 (1.028, 1.085)	< 0.001	PM_2.5_	1.203 (1.178, 1.230)	< 0.001
PM_10_	1.029 (1.002, 1.057)	0.034	PM_10_	1.049 (1.020, 1.079)	< 0.001
NO_2_	1.045 (1.017, 1.074)	0.001	NO_2_	1.133 (1.105, 1.163)	< 0.001
NO_x_	1.051 (1.023, 1.080)	< 0.001	NO_x_	1.168 (1.145, 1.192)	< 0.001

HR and 95% CI were estimated using weighted Cox regression. Model adjusted for age, sex, ethnicity, education levels, employment status, household income, body mass index, physical activity, smoking status, alcohol intake, healthy diet pattern, cardiovascular diseases history, diabetes history, hypertension history, estimated glomerular filtration rate, low-density lipoprotein, natural logarithm of triglycerides.

### 3.5 . Metabolic signatures partially mediate the association between air pollution and RA risk

**Figure 3:**
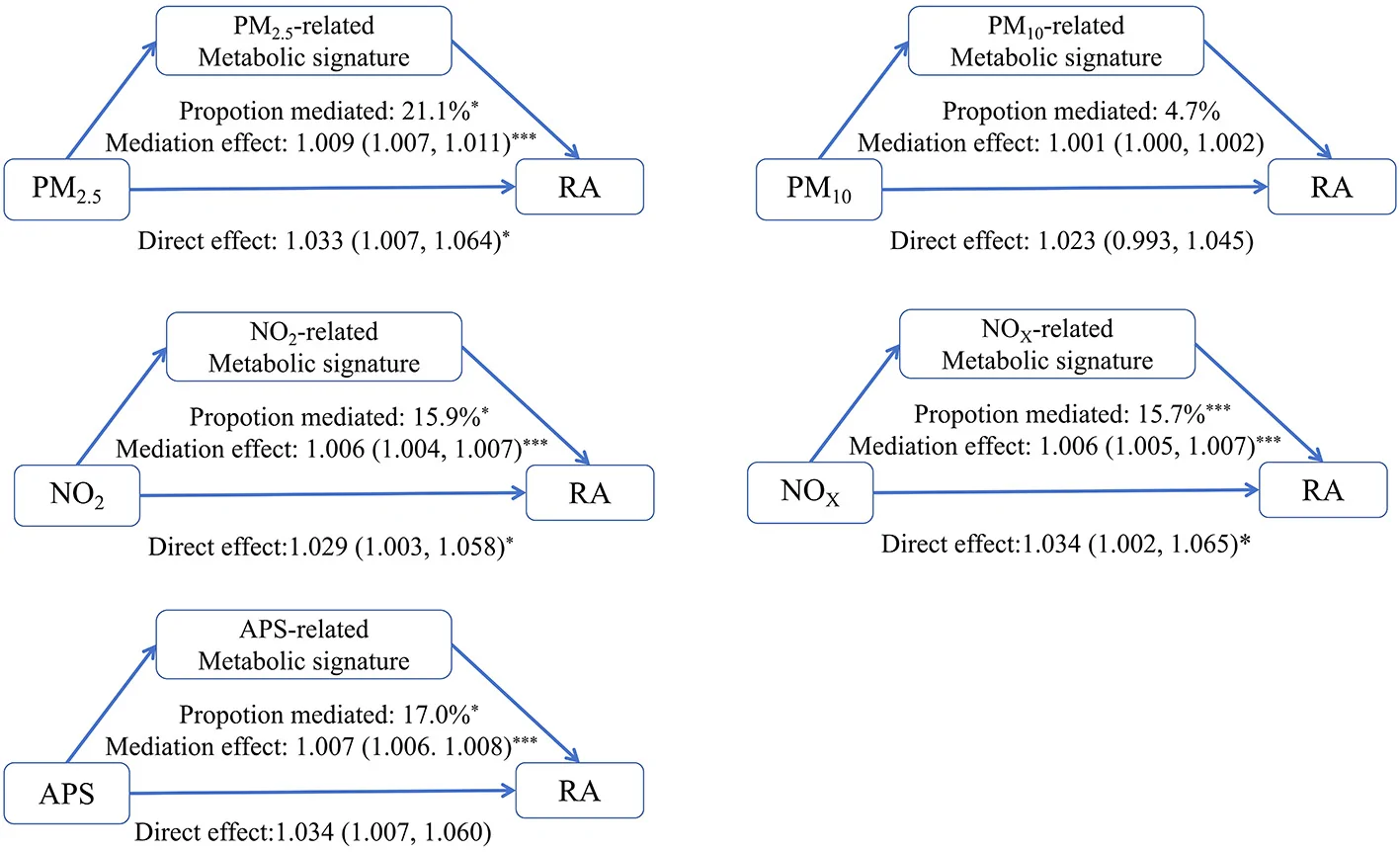
Mediation analyses of metabolic signatures between air pollutants and rheumatoid arthritis. Model adjusted for age, sex, ethnicity, education levels, employment status, household income, body mass index, physical activity, smoking status, alcohol intake, healthy diet pattern, cardiovascular diseases history, diabetes history, hypertension history, estimated glomerular filtration rate, low-density lipoprotein, natural logarithm of triglycerides. ^*^P < 0.05, ^***^P < 0.001.

We suggested that ${\text{PM}}_{2.5^{-}}$, ${\text{NO}}_{2^{-}}$, ${\text{NO}}_{{\text{x}}^{-}}$ and APS-related metabolic signature partly explained the associations between air pollutants or APS and RA risk the associations of air pollutants or APS with RA risk (Figure 3). The estimated proportions explained by metabolic signatures were 21.1% (95% CI: 12.6%, 43.1%) for PM_2.5_, 15.9% (95% CI: 8.0%, 52.9%) for NO_2_, 15.7% (95% CI: 9.5%, 58.3%) for NO_x_, and 17.0% (95% CI: 10.2%, 41.6%) for APS. No significant additive or multiplicative effects were found between air pollutants or APS and related metabolic signatures (Supplementary Table S5).

### 3.6 . Sensitivity analyses

The observed associations remained robust across a series of sensitivity analyses. Specifically, the results were not materially altered when long-term exposure was assessed using a time-varying Cox regression model (Supplementary Table S6), competing risks were accounted for using a Fine–Gray model (Supplementary Table S7), potential bias from unmeasured confounding was evaluated using E-values (Supplementary Table S8), or analyses were repeated using data after multiple imputation (Supplementary Table S9). Likewise, additional adjustment for fasting time (Supplementary Table S10) or natural environment, green space, and water space (Supplementary Table S11), exclusion of RA events at baseline and within the first 1 or 2 years of follow-up (Supplementary Table S12), use of alternative exposure windows of one, two, and 3 years for air pollutants (Supplementary Table S13), and exclusion of LDL cholesterol and natural log-transformed triglycerides from the covariate set (Supplementary Table S14) did not materially change the results. In addition, an equal-weighted APS yielded a similar positive association with incident RA risk (Supplementary Table S15).

## 4 Discussion

This large prospective study provided evidence that plasma metabolic signatures related to PM_2.5_, NO_2_, NO_x_, and APS are associated with an increased risk of RA and may mediate approximately 15.7% to 21.1% of the associations between specific air pollutants and RA risk. Collectively, these findings support a plausible metabolic mechanism linking air pollution to RA onset and highlight potential targets for early prevention and intervention.

Our observations align with a growing body of epidemiological studies suggesting a role for air pollution in RA pathogenesis. A large-scale cohort study of 342,973 participants found that the APS, representing the combined exposure to multiple air pollutants, was associated with an increased risk of incident RA, particularly among individuals with high genetic susceptibility (6). Similarly, a national cohort in South Korea including 230,034 participants indicated that PM_2.5_, but not coarse particulate matter, was associated with autoimmune rheumatic disease risk (29). A large Canadian cohort study by Zhao et al. further suggested that PM_2.5_ chemical components may be associated with RA risk, indicating that particulate composition, rather than total particulate mass alone, may be relevant to RA-related immune responses (14). For various air pollutants emission sources, a case–control study comprising 9,701 incident RA cases and 68,852 matched controls showed that fire smoke-derived PM_2.5_ was associated with RA-associated interstitial lung disease (RA-ILD), while NO_x_ and PM_10_ were associated with an increased risk of RA (30). Besides, a longitudinal study from national health insurance datasets has linked exposure to traffic-related pollutants such as NO_2_ with a higher risk of RA (31). The evidence remains mixed, such as the longitudinal design from Nurses' Health Study and the case-control design from the Swedish EIRA, observe inconsistent associations between exposure to air pollution and RA risk (12, 13). These discrepancies may be attributable to variations in exposure assessment, study populations, follow-up duration, and genetic or environmental context (33). Our findings from a large, well-characterized cohort with detailed exposure assessment and long-term follow-up provide robust contemporary support for a positive association, particularly for PM_2.5_ and nitrogen oxides.

Our findings demonstrated that long-term exposure to air pollutants was associated with broad perturbations in the plasma metabolome. The observed metabolic perturbations were largely concentrated in lipoprotein lipids, fatty acids, and amino acids, aligning with earlier studies that reported broad metabolic responses to air pollution exposure (34, 35). Population-based evidence has suggested that exposure to air pollutants may influence circulating lipid profiles, including alterations in apolipoproteins and high-density lipoprotein (HDL) subclasses, as well as perturbations in amino acid metabolism (34, 35). For example, evidence from 82,548 diabetes-free participants indicated that air pollution may induce a metabolomic signature characterized by higher apolipoprotein A1 concentrations and larger HDL particles, and this signature partly explained the observed association between air pollution exposure and incident type 2 diabetes (34). In addition, an untargeted metabolomic study that involved 31 healthy volunteers identified tyrosine and guanosine as blood metabolites associated with short-term air pollution exposure (32). Pathway enrichment analysis further indicated that these metabolic alterations were concentrated in glyoxylate and dicarboxylate metabolism and in the biosynthesis and metabolism of phenylalanine, tyrosine, and tryptophan. These metabolic signatures significantly mediated the associations of PM_2.5_, NO_2_, NO_x_, and APS with incident RA, accounting for 21.1%, 15.9%, 15.7%, and 17.0% of the associations, respectively. Collectively, these findings support a plausible air pollution–metabolism–RA axis and suggest that systemic metabolic dysregulation may represent one potential mechanism through which long-term air pollution exposure contributes to RA development (36).

Our findings provided evidence for an intermediate link between air pollution exposure and RA risk. Air pollution exposure, particularly PM_2.5_, can induce systemic inflammation and oxidative stress through metabolic dysregulation, both of which play central roles in the initiation and perpetuation of autoimmune responses (37, 38). The multi-omics study by Zhao et al. suggested that PM_10_ exposure may be linked to immune-cell dysfunction and RA progression, highlighting the potential role of air pollution in reshaping immune and inflammatory networks (16). Particulate matter may also contribute to immunotoxicity, at least in part, through mitochondrial dysfunction. Disruption of mitochondrial redox homeostasis and metabolic activity may generate damage-associated molecular patterns, which can activate innate immune pathways, including the NLRP3 inflammasome and cGAS–STING signaling (39). The inflammatory milieu may originate in the lungs, where chronic inflammatory stimulation can promote the formation of inducible bronchus-associated lymphoid tissue (iBALT). This immune activation may facilitate protein citrullination, a post-translational modification closely implicated in RA-related autoimmunity (40, 41). The metabolomic disturbances we identified may both reflect and amplify these processes. Pollution-induced oxidative stress may produce pro-inflammatory lipids, including cytochrome P450-derived species, that mediate responses to particulate matter in susceptible individuals (42). These altered lipids and metabolites may act as neoantigens or impair immune tolerance, contributing to the loss of self-tolerance characteristic of RA (43, 44).

Our stratified analyses showed stronger associations of PM_10_, NO_2_, and NO_x_ with RA risk in women than in men, underscoring the importance of sex in environmental risk assessment. Although the underlying mechanisms remain to be clarified, hormonal factors, particularly estrogen-mediated immune modulation, together with differences in inflammatory status, body composition, and exposure patterns, may contribute (45–47). Compared with men, women exhibit a more pronounced pro-inflammatory immune profile, characterized by enhanced lymphocyte proliferation and activation and increased production of pro-inflammatory cytokines, whereas men more often display expansion of regulatory cell subsets (47). This difference may partly explain why women are more susceptible to air pollution-induced inflammatory and immune responses (47). Further research is warranted to elucidate these mechanisms.

Several limitations warrant discussion. First, despite adjustment for a broad range of covariates, residual confounding from unmeasured or imperfectly measured factors, such as occupational exposure, traffic noise, area-level deprivation, passive smoking, family history of RA, genetic susceptibility, and other environmental or behavioral factors, may have influenced the observed associations. Second, exposure assessment was based on residential outdoor air pollution estimates, which may not fully capture personal exposure or indoor air pollution levels, potentially resulting in exposure misclassification. Third, plasma metabolomic profiles were measured only once at baseline, and the timing of metabolomic sample collection did not fully correspond to all exposure assessment windows. Although the 2010 annual average air pollution estimates were used to represent long-term residential exposure and sensitivity analyses using time-varying and alternative exposure windows yielded similar results, some degree of exposure misclassification remains possible, and the temporal ordering between air pollution exposure and metabolic alterations could not be fully established. Therefore, the mediation findings should be interpreted as exploratory evidence of potential biological pathways rather than definitive causal mediation. Fourth, because the study population was predominantly of European ancestry, the generalizability of our findings to other ethnic groups or geographic regions may be limited, particularly in populations with different genetic backgrounds, air pollution characteristics, and lifestyle patterns. Fifth, because the APS weights were derived from the same study population, some degree of overfitting and limited external transportability of the weighted score cannot be excluded. Therefore, the APS should be interpreted as a study-specific composite indicator of air pollution burden, and external validation in independent cohorts is warranted. Sixth, although GPS weighting was used to improve covariate balance, this approach can only account for measured covariates included in the weighting model and cannot eliminate residual or unmeasured confounding. Therefore, the weighted analyses should be interpreted as robustness analyses of associations rather than evidence of causality.

## 5 Conclusion

In conclusion, we identified metabolic signatures related to PM_2.5_, NO_2_, NO_x_, and APS, and these signatures were associated with a higher risk of incident RA. Mediation analyses further suggested that metabolic alterations partly explained the relationships between air pollution exposure and RA risk. These results highlight metabolomic perturbations as potential biological links between ambient air pollution and RA development, and may inform future strategies for autoimmune disease prevention.

## Data Availability

The data analyzed in this study is subject to the following licenses/restrictions: The data used in the present study are available from the UK Biobank with restrictions applied. Access to the UK Biobank data can be requested by means of a standard protocol (https://www.ukbiobank.ac.uk/register-apply/). Requests to access these datasets should be directed to https://www.ukbiobank.ac.uk/register-apply/.
