# Supplementary material for: Plasma metabolomic signatures of air pollution and rheumatoid arthritis risk
Source: Front Public Health. 2026 Aug 12;14:1901951. doi: 10.3389/fpubh.2026.1901951 (PMC13506800; doi:10.3389/fpubh.2026.1901951)
Supplement: Supplementary file 1 [file Supplementary_file_1.docx]

***Supplementary Files***

**Plasma metabolomic signatures of air pollution and rheumatoid arthritis risk**

Yangchang Zhang, Liang Tian, Yang Pu, Linpeng Zhou, Lingli Ding, Chengyin Li, Xun Lei, Bin Wu

**Table S1.** The detailed information of included metabolites in the study.

**Table S2.** Summary statistics of air pollutants.

**Table S3.** Pathway Analysis of air pollutant score-related metabolites.

**Table S4.** Associations between pollutant mixture exposure and incident rheumatoid arthritis using quantile g-computation models.

**Table S5.** The additive interactions between metabolic signature and ambient air pollution on the risk of rheumatoid arthritis.

**Table S6.** Associations of air pollution with risk of rheumatoid arthritis using time-varying Cox regression model.

**Table S7.** Associations of air pollution with risk of rheumatoid arthritis using Fine-Gray model.

**Table S8.** The associations of air pollution and related metabolic signatures with rheumatoid arthritis using E-value estimates.

**Table S9.** Associations of air pollutants and their related metabolic signatures with rheumatoid arthritis after multiple imputation of missing data.

**Table S10.** Association of air pollutants and related metabolic signatures with rheumatoid arthritis including fasting time for adjustment.

**Table S11.** Associations of air pollutants and their related metabolic signatures with rheumatoid arthritis after adjusted for natural environment, green space, and water space.

**Table S12.** Associations of air pollutants and their related metabolic signatures with rheumatoid arthritis after excluding events at baseline and within the first 1 and 2 years of follow-up.

**Table S13.** Association of air pollutants with rheumatoid arthritis using the 1-year, 2-year and 3-year exposure window.

**Table S14.** Associations of air pollutants and their related metabolic signatures with rheumatoid arthritis after excluding LDL cholesterol and triglycerides from the covariate set.

**Table S15.** Associations between equal-weighted air pollution score and rheumatoid arthritis risk.

**Figure S1.** Flowchart of participant selection for the study.

**Figure S2.** Direct acyclic graph of the association between air pollution and rheumatoid arthritis risk.

**Figure S3.** Associations of metabolites comprising the PM_2.5_-related metabolic signature with rheumatoid arthritis and air pollutants.

**Figure S4.** Associations of metabolites comprising the PM_10_-related metabolic signature with rheumatoid arthritis and air pollutants.

**Figure S5.** Associations of metabolites comprising the NO_2_-related metabolic signature with rheumatoid arthritis and air pollutants.

**Figure S6.** Associations of metabolites comprising the NO_x_-related metabolic signature with rheumatoid arthritis and air pollutants.

**Figure S7.** The composition of air pollution-related metabolic signatures.

**Figure S8.** Subgroup analyses for the association of air pollutants with rheumatoid arthritis.

**Figure S9.** Exposure–response relationships of air pollution and related metabolic signatures with rheumatoid arthritis risk based on restricted cubic spline models with knots at the 10th, 50th, and 90th percentiles.

**Supplementary Methods**

**Table S1.** The detailed information of included metabolites in the study.

| Field id | Metabolite | Abbreviation | Group | Unit |
| --- | --- | --- | --- | --- |
| 23474 | 3-Hydroxybutyrate | bOHbutyrate | Ketone bodies | mmol/l |
| 23475 | Acetate | Acetate | Ketone bodies | mmol/l |
| 23476 | Acetoacetate | Acetoacetate | Ketone bodies | mmol/l |
| 23477 | Acetone | Acetone | Ketone bodies | mmol/l |
| 23460 | Alanine | Alanine | Amino acids | mmol/l |
| 23479 | Albumin | Albumin | Fluid balance | g/l |
| 23479 | Albumin | Albumin | Fluid balance | g/l |
| 23440 | Apolipoprotein A1 | ApoA1 | Apolipoproteins | g/l |
| 23439 | Apolipoprotein B | ApoB | Apolipoproteins | g/l |
| 23439 | Apolipoprotein B | ApoB | Apolipoproteins | g/l |
| 23433 | Average diameter for HDL particles | HDL particle size | Lipoprotein particle sizes | nm |
| 23432 | Average diameter for LDL particles | LDL particle size | Lipoprotein particle sizes | nm |
| 23431 | Average diameter for VLDL particles | VLDL particle size | Lipoprotein particle sizes | nm |
| 23484 | Cholesterol in chylomicrons and extremely large VLDL | XXL-VLDL-C | Lipoprotein subclasses | mmol/l |
| 23526 | Cholesterol in IDL | IDL-C | Lipoprotein subclasses | mmol/l |
| 23561 | Cholesterol in large HDL | L-HDL-C | Lipoprotein subclasses | mmol/l |
| 23533 | Cholesterol in large LDL | L-LDL-C | Lipoprotein subclasses | mmol/l |
| 23498 | Cholesterol in large VLDL | L-VLDL-C | Lipoprotein subclasses | mmol/l |
| 23568 | Cholesterol in medium HDL | M-HDL-C | Lipoprotein subclasses | mmol/l |
| 23540 | Cholesterol in medium LDL | M-LDL-C | Lipoprotein subclasses | mmol/l |
| 23505 | Cholesterol in medium VLDL | M-VLDL-C | Lipoprotein subclasses | mmol/l |
| 23575 | Cholesterol in small HDL | S-HDL-C | Lipoprotein subclasses | mmol/l |
| 23547 | Cholesterol in small LDL | S-LDL-C | Lipoprotein subclasses | mmol/l |
| 23512 | Cholesterol in small VLDL | S-VLDL-C | Lipoprotein subclasses | mmol/l |
| 23554 | Cholesterol in very large HDL | XL-HDL-C | Lipoprotein subclasses | mmol/l |
| 23491 | Cholesterol in very large VLDL | XL-VLDL-C | Lipoprotein subclasses | mmol/l |
| 23519 | Cholesterol in very small VLDL | XS-VLDL-C | Lipoprotein subclasses | mmol/l |
| 23580 | Cholesterol to total lipids ratio in chylomicrons and extremely large VLDL | XXL-VLDL-C % | Relative lipoprotein lipid concentrations | % |
| 23610 | Cholesterol to total lipids ratio in IDL | IDL-C % | Relative lipoprotein lipid concentrations | % |
| 23635 | Cholesterol to total lipids ratio in large HDL | L-HDL-C % | Relative lipoprotein lipid concentrations | % |
| 23615 | Cholesterol to total lipids ratio in large LDL | L-LDL-C % | Relative lipoprotein lipid concentrations | % |
| 23590 | Cholesterol to total lipids ratio in large VLDL | L-VLDL-C % | Relative lipoprotein lipid concentrations | % |
| 23640 | Cholesterol to total lipids ratio in medium HDL | M-HDL-C % | Relative lipoprotein lipid concentrations | % |
| 23620 | Cholesterol to total lipids ratio in medium LDL | M-LDL-C % | Relative lipoprotein lipid concentrations | % |
| 23595 | Cholesterol to total lipids ratio in medium VLDL | M-VLDL-C % | Relative lipoprotein lipid concentrations | % |
| 23645 | Cholesterol to total lipids ratio in small HDL | S-HDL-C % | Relative lipoprotein lipid concentrations | % |
| 23625 | Cholesterol to total lipids ratio in small LDL | S-LDL-C % | Relative lipoprotein lipid concentrations | % |
| 23600 | Cholesterol to total lipids ratio in small VLDL | S-VLDL-C % | Relative lipoprotein lipid concentrations | % |
| 23630 | Cholesterol to total lipids ratio in very large HDL | XL-HDL-C % | Relative lipoprotein lipid concentrations | % |
| 23585 | Cholesterol to total lipids ratio in very large VLDL | XL-VLDL-C % | Relative lipoprotein lipid concentrations | % |
| 23605 | Cholesterol to total lipids ratio in very small VLDL | XS-VLDL-C % | Relative lipoprotein lipid concentrations | % |
| 23485 | Cholesteryl esters in chylomicrons and extremely large VLDL | XXL-VLDL-CE | Lipoprotein subclasses | mmol/l |
| 23418 | Cholesteryl esters in HDL | HDL-CE | Cholesteryl esters | mmol/l |
| 23527 | Cholesteryl esters in IDL | IDL-CE | Lipoprotein subclasses | mmol/l |
| 23562 | Cholesteryl esters in large HDL | L-HDL-CE | Lipoprotein subclasses | mmol/l |
| 23534 | Cholesteryl esters in large LDL | L-LDL-CE | Lipoprotein subclasses | mmol/l |
| 23499 | Cholesteryl esters in large VLDL | L-VLDL-CE | Lipoprotein subclasses | mmol/l |
| 23417 | Cholesteryl esters in LDL | LDL-CE | Cholesteryl esters | mmol/l |
| 23569 | Cholesteryl esters in medium HDL | M-HDL-CE | Lipoprotein subclasses | mmol/l |
| 23541 | Cholesteryl esters in medium LDL | M-LDL-CE | Lipoprotein subclasses | mmol/l |
| 23506 | Cholesteryl esters in medium VLDL | M-VLDL-CE | Lipoprotein subclasses | mmol/l |
| 23576 | Cholesteryl esters in small HDL | S-HDL-CE | Lipoprotein subclasses | mmol/l |
| 23548 | Cholesteryl esters in small LDL | S-LDL-CE | Lipoprotein subclasses | mmol/l |
| 23513 | Cholesteryl esters in small VLDL | S-VLDL-CE | Lipoprotein subclasses | mmol/l |
| 23555 | Cholesteryl esters in very large HDL | XL-HDL-CE | Lipoprotein subclasses | mmol/l |
| 23492 | Cholesteryl esters in very large VLDL | XL-VLDL-CE | Lipoprotein subclasses | mmol/l |
| 23520 | Cholesteryl esters in very small VLDL | XS-VLDL-CE | Lipoprotein subclasses | mmol/l |
| 23416 | Cholesteryl esters in VLDL | VLDL-CE | Cholesteryl esters | mmol/l |
| 23581 | Cholesteryl esters to total lipids ratio in chylomicrons and extremely large VLDL | XXL-VLDL-CE % | Relative lipoprotein lipid concentrations | % |
| 23611 | Cholesteryl esters to total lipids ratio in IDL | IDL-CE % | Relative lipoprotein lipid concentrations | % |
| 23636 | Cholesteryl esters to total lipids ratio in large HDL | L-HDL-CE % | Relative lipoprotein lipid concentrations | % |
| 23616 | Cholesteryl esters to total lipids ratio in large LDL | L-LDL-CE % | Relative lipoprotein lipid concentrations | % |
| 23591 | Cholesteryl esters to total lipids ratio in large VLDL | L-VLDL-CE % | Relative lipoprotein lipid concentrations | % |
| 23641 | Cholesteryl esters to total lipids ratio in medium HDL | M-HDL-CE % | Relative lipoprotein lipid concentrations | % |
| 23621 | Cholesteryl esters to total lipids ratio in medium LDL | M-LDL-CE % | Relative lipoprotein lipid concentrations | % |
| 23596 | Cholesteryl esters to total lipids ratio in medium VLDL | M-VLDL-CE % | Relative lipoprotein lipid concentrations | % |
| 23646 | Cholesteryl esters to total lipids ratio in small HDL | S-HDL-CE % | Relative lipoprotein lipid concentrations | % |
| 23626 | Cholesteryl esters to total lipids ratio in small LDL | S-LDL-CE % | Relative lipoprotein lipid concentrations | % |
| 23601 | Cholesteryl esters to total lipids ratio in small VLDL | S-VLDL-CE % | Relative lipoprotein lipid concentrations | % |
| 23631 | Cholesteryl esters to total lipids ratio in very large HDL | XL-HDL-CE % | Relative lipoprotein lipid concentrations | % |
| 23586 | Cholesteryl esters to total lipids ratio in very large VLDL | XL-VLDL-CE % | Relative lipoprotein lipid concentrations | % |
| 23606 | Cholesteryl esters to total lipids ratio in very small VLDL | XS-VLDL-CE % | Relative lipoprotein lipid concentrations | % |
| 23473 | Citrate | Citrate | Glycolysis related metabolites | mmol/l |
| 23404 | Clinical LDL cholesterol | Clinical LDL-C | Cholesterol | mmol/l |
| 23481 | Concentration of chylomicrons and extremely large VLDL particles | XXL-VLDL-P | Lipoprotein subclasses | mmol/l |
| 23430 | Concentration of HDL particles | HDL-P | Lipoprotein particle concentrations | mmol/l |
| 23523 | Concentration of IDL particles | IDL-P | Lipoprotein subclasses | mmol/l |
| 23558 | Concentration of large HDL particles | L-HDL-P | Lipoprotein subclasses | mmol/l |
| 23530 | Concentration of large LDL particles | L-LDL-P | Lipoprotein subclasses | mmol/l |
| 23495 | Concentration of large VLDL particles | L-VLDL-P | Lipoprotein subclasses | mmol/l |
| 23429 | Concentration of LDL particles | LDL-P | Lipoprotein particle concentrations | mmol/l |
| 23565 | Concentration of medium HDL particles | M-HDL-P | Lipoprotein subclasses | mmol/l |
| 23537 | Concentration of medium LDL particles | M-LDL-P | Lipoprotein subclasses | mmol/l |
| 23502 | Concentration of medium VLDL particles | M-VLDL-P | Lipoprotein subclasses | mmol/l |
| 23572 | Concentration of small HDL particles | S-HDL-P | Lipoprotein subclasses | mmol/l |
| 23544 | Concentration of small LDL particles | S-LDL-P | Lipoprotein subclasses | mmol/l |
| 23509 | Concentration of small VLDL particles | S-VLDL-P | Lipoprotein subclasses | mmol/l |
| 23551 | Concentration of very large HDL particles | XL-HDL-P | Lipoprotein subclasses | mmol/l |
| 23488 | Concentration of very large VLDL particles | XL-VLDL-P | Lipoprotein subclasses | mmol/l |
| 23516 | Concentration of very small VLDL particles | XS-VLDL-P | Lipoprotein subclasses | mmol/l |
| 23428 | Concentration of VLDL particles | VLDL-P | Lipoprotein particle concentrations | mmol/l |
| 23478 | Creatinine | Creatinine | Fluid balance | mmol/l |
| 23478 | Creatinine | Creatinine | Fluid balance | umol/l |
| 23443 | Degree of unsaturation | Unsaturation | Fatty acids | degree |
| 23450 | Docosahexaenoic acid | DHA | Fatty acids | mmol/l |
| 23486 | Free cholesterol in chylomicrons and extremely large VLDL | XXL-VLDL-FC | Lipoprotein subclasses | mmol/l |
| 23422 | Free cholesterol in HDL | HDL-FC | Free cholesterol | mmol/l |
| 23528 | Free cholesterol in IDL | IDL-FC | Lipoprotein subclasses | mmol/l |
| 23563 | Free cholesterol in large HDL | L-HDL-FC | Lipoprotein subclasses | mmol/l |
| 23535 | Free cholesterol in large LDL | L-LDL-FC | Lipoprotein subclasses | mmol/l |
| 23500 | Free cholesterol in large VLDL | L-VLDL-FC | Lipoprotein subclasses | mmol/l |
| 23421 | Free cholesterol in LDL | LDL-FC | Free cholesterol | mmol/l |
| 23570 | Free cholesterol in medium HDL | M-HDL-FC | Lipoprotein subclasses | mmol/l |
| 23542 | Free cholesterol in medium LDL | M-LDL-FC | Lipoprotein subclasses | mmol/l |
| 23507 | Free cholesterol in medium VLDL | M-VLDL-FC | Lipoprotein subclasses | mmol/l |
| 23577 | Free cholesterol in small HDL | S-HDL-FC | Lipoprotein subclasses | mmol/l |
| 23549 | Free cholesterol in small LDL | S-LDL-FC | Lipoprotein subclasses | mmol/l |
| 23514 | Free cholesterol in small VLDL | S-VLDL-FC | Lipoprotein subclasses | mmol/l |
| 23556 | Free cholesterol in very large HDL | XL-HDL-FC | Lipoprotein subclasses | mmol/l |
| 23493 | Free cholesterol in very large VLDL | XL-VLDL-FC | Lipoprotein subclasses | mmol/l |
| 23521 | Free cholesterol in very small VLDL | XS-VLDL-FC | Lipoprotein subclasses | mmol/l |
| 23420 | Free cholesterol in VLDL | VLDL-FC | Free cholesterol | mmol/l |
| 23582 | Free cholesterol to total lipids ratio in chylomicrons and extremely large VLDL | XXL-VLDL-FC % | Relative lipoprotein lipid concentrations | % |
| 23612 | Free cholesterol to total lipids ratio in IDL | IDL-FC % | Relative lipoprotein lipid concentrations | % |
| 23637 | Free cholesterol to total lipids ratio in large HDL | L-HDL-FC % | Relative lipoprotein lipid concentrations | % |
| 23617 | Free cholesterol to total lipids ratio in large LDL | L-LDL-FC % | Relative lipoprotein lipid concentrations | % |
| 23592 | Free cholesterol to total lipids ratio in large VLDL | L-VLDL-FC % | Relative lipoprotein lipid concentrations | % |
| 23642 | Free cholesterol to total lipids ratio in medium HDL | M-HDL-FC % | Relative lipoprotein lipid concentrations | % |
| 23622 | Free cholesterol to total lipids ratio in medium LDL | M-LDL-FC % | Relative lipoprotein lipid concentrations | % |
| 23597 | Free cholesterol to total lipids ratio in medium VLDL | M-VLDL-FC % | Relative lipoprotein lipid concentrations | % |
| 23647 | Free cholesterol to total lipids ratio in small HDL | S-HDL-FC % | Relative lipoprotein lipid concentrations | % |
| 23627 | Free cholesterol to total lipids ratio in small LDL | S-LDL-FC % | Relative lipoprotein lipid concentrations | % |
| 23602 | Free cholesterol to total lipids ratio in small VLDL | S-VLDL-FC % | Relative lipoprotein lipid concentrations | % |
| 23632 | Free cholesterol to total lipids ratio in very large HDL | XL-HDL-FC % | Relative lipoprotein lipid concentrations | % |
| 23587 | Free cholesterol to total lipids ratio in very large VLDL | XL-VLDL-FC % | Relative lipoprotein lipid concentrations | % |
| 23607 | Free cholesterol to total lipids ratio in very small VLDL | XS-VLDL-FC % | Relative lipoprotein lipid concentrations | % |
| 23470 | Glucose | Glucose | Glycolysis related metabolites | mmol/l |
| 23470 | Glucose | Glucose | Glycolysis related metabolites | mmol/l |
| 20280 | Glucose-lactate | GL | Glycolysis related metabolites | mmol/l |
| 23461 | Glutamine | Glutamine | Amino acids | mmol/l |
| 23462 | Glycine | Glycine | Amino acids | mmol/l |
| 23480 | Glycoprotein acetyls | Glycoprotein acetyls | Inflammation | mmol/l |
| 23406 | HDL cholesterol | HDL-C | Cholesterol | mmol/l |
| 23463 | Histidine | Histidine | Amino acids | mmol/l |
| 23465 | Isoleucine | Isoleucine | Amino acids | mmol/l |
| 23471 | Lactate | Lactate | Glycolysis related metabolites | mmol/l |
| 23405 | LDL cholesterol | LDL-C | Cholesterol | mmol/l |
| 23405 | LDL cholesterol | LDL-C | Cholesterol | mmol/l |
| 23466 | Leucine | Leucine | Amino acids | mmol/l |
| 23449 | Linoleic acid | LA | Fatty acids | mmol/l |
| 23447 | Monounsaturated fatty acids | MUFA | Fatty acids | mmol/l |
| 23444 | Omega-3 fatty acids | Omega-3 | Fatty acids | mmol/l |
| 23445 | Omega-6 fatty acids | Omega-6 | Fatty acids | mmol/l |
| 23468 | Phenylalanine | Phenylalanine | Amino acids | mmol/l |
| 23437 | Phosphatidylcholines | Phosphatidylcholines | Other lipids | mmol/l |
| 23434 | Phosphoglycerides | Phosphoglycerides | Other lipids | mmol/l |
| 23483 | Phospholipids in chylomicrons and extremely large VLDL | XXL-VLDL-PL | Lipoprotein subclasses | mmol/l |
| 23414 | Phospholipids in HDL | HDL-PL | Phospholipids | mmol/l |
| 23525 | Phospholipids in IDL | IDL-PL | Lipoprotein subclasses | mmol/l |
| 23560 | Phospholipids in large HDL | L-HDL-PL | Lipoprotein subclasses | mmol/l |
| 23532 | Phospholipids in large LDL | L-LDL-PL | Lipoprotein subclasses | mmol/l |
| 23497 | Phospholipids in large VLDL | L-VLDL-PL | Lipoprotein subclasses | mmol/l |
| 23413 | Phospholipids in LDL | LDL-PL | Phospholipids | mmol/l |
| 23567 | Phospholipids in medium HDL | M-HDL-PL | Lipoprotein subclasses | mmol/l |
| 23539 | Phospholipids in medium LDL | M-LDL-PL | Lipoprotein subclasses | mmol/l |
| 23504 | Phospholipids in medium VLDL | M-VLDL-PL | Lipoprotein subclasses | mmol/l |
| 23574 | Phospholipids in small HDL | S-HDL-PL | Lipoprotein subclasses | mmol/l |
| 23546 | Phospholipids in small LDL | S-LDL-PL | Lipoprotein subclasses | mmol/l |
| 23511 | Phospholipids in small VLDL | S-VLDL-PL | Lipoprotein subclasses | mmol/l |
| 23553 | Phospholipids in very large HDL | XL-HDL-PL | Lipoprotein subclasses | mmol/l |
| 23490 | Phospholipids in very large VLDL | XL-VLDL-PL | Lipoprotein subclasses | mmol/l |
| 23518 | Phospholipids in very small VLDL | XS-VLDL-PL | Lipoprotein subclasses | mmol/l |
| 23412 | Phospholipids in VLDL | VLDL-PL | Phospholipids | mmol/l |
| 23579 | Phospholipids to total lipids ratio in chylomicrons and extremely large VLDL | XXL-VLDL-PL % | Relative lipoprotein lipid concentrations | % |
| 23609 | Phospholipids to total lipids ratio in IDL | IDL-PL % | Relative lipoprotein lipid concentrations | % |
| 23634 | Phospholipids to total lipids ratio in large HDL | L-HDL-PL % | Relative lipoprotein lipid concentrations | % |
| 23614 | Phospholipids to total lipids ratio in large LDL | L-LDL-PL % | Relative lipoprotein lipid concentrations | % |
| 23589 | Phospholipids to total lipids ratio in large VLDL | L-VLDL-PL % | Relative lipoprotein lipid concentrations | % |
| 23639 | Phospholipids to total lipids ratio in medium HDL | M-HDL-PL % | Relative lipoprotein lipid concentrations | % |
| 23619 | Phospholipids to total lipids ratio in medium LDL | M-LDL-PL % | Relative lipoprotein lipid concentrations | % |
| 23594 | Phospholipids to total lipids ratio in medium VLDL | M-VLDL-PL % | Relative lipoprotein lipid concentrations | % |
| 23644 | Phospholipids to total lipids ratio in small HDL | S-HDL-PL % | Relative lipoprotein lipid concentrations | % |
| 23624 | Phospholipids to total lipids ratio in small LDL | S-LDL-PL % | Relative lipoprotein lipid concentrations | % |
| 23599 | Phospholipids to total lipids ratio in small VLDL | S-VLDL-PL % | Relative lipoprotein lipid concentrations | % |
| 23629 | Phospholipids to total lipids ratio in very large HDL | XL-HDL-PL % | Relative lipoprotein lipid concentrations | % |
| 23584 | Phospholipids to total lipids ratio in very large VLDL | XL-VLDL-PL % | Relative lipoprotein lipid concentrations | % |
| 23604 | Phospholipids to total lipids ratio in very small VLDL | XS-VLDL-PL % | Relative lipoprotein lipid concentrations | % |
| 23446 | Polyunsaturated fatty acids | PUFA | Fatty acids | mmol/l |
| 23472 | Pyruvate | Pyruvate | Glycolysis related metabolites | mmol/l |
| 23441 | Ratio of apolipoprotein B to apolipoprotein A1 | ApoB/ApoA1 | Apolipoproteins | ratio |
| 23457 | Ratio of docosahexaenoic acid to total fatty acids | DHA % | Fatty acids | % |
| 23456 | Ratio of linoleic acid to total fatty acids | LA % | Fatty acids | % |
| 23454 | Ratio of monounsaturated fatty acids to total fatty acids | MUFA % | Fatty acids | % |
| 23451 | Ratio of omega-3 fatty acids to total fatty acids | Omega-3 % | Fatty acids | % |
| 23459 | Ratio of omega-6 fatty acids to omega-3 fatty acids | Omega-6/Omega-3 | Fatty acids | ratio |
| 23452 | Ratio of omega-6 fatty acids to total fatty acids | Omega-6 % | Fatty acids | % |
| 23458 | Ratio of polyunsaturated fatty acids to monounsaturated fatty acids | PUFA/MUFA | Fatty acids | ratio |
| 23453 | Ratio of polyunsaturated fatty acids to total fatty acids | PUFA % | Fatty acids | % |
| 23455 | Ratio of saturated fatty acids to total fatty acids | SFA % | Fatty acids | % |
| 23435 | Ratio of triglycerides to phosphoglycerides | TG/PG | Other lipids | ratio |
| 23402 | Remnant cholesterol (non-HDL, non-LDL -cholesterol) | Remnant-C | Cholesterol | mmol/l |
| 23448 | Saturated fatty acids | SFA | Fatty acids | mmol/l |
| 20281 | Spectrometer-corrected alanine | SCA | Amino acids | mmol/l |
| 23438 | Sphingomyelins | Sphingomyelins | Other lipids | mmol/l |
| 23400 | Total cholesterol | Total-C | Cholesterol | mmol/l |
| 23401 | Total cholesterol minus HDL-C | non-HDL-C | Cholesterol | mmol/l |
| 23436 | Total cholines | Total cholines | Other lipids | mmol/l |
| 23464 | Total concentration of branched-chain amino acids (leucine + isoleucine + valine) | Total BCAA | Amino acids | mmol/l |
| 23427 | Total concentration of lipoprotein particles | Total-P | Lipoprotein particle concentrations | mmol/l |
| 23415 | Total esterified cholesterol | Total-CE | Cholesteryl esters | mmol/l |
| 23442 | Total fatty acids | Total fatty acids | Fatty acids | mmol/l |
| 23419 | Total free cholesterol | Total-FC | Free cholesterol | mmol/l |
| 23482 | Total lipids in chylomicrons and extremely large VLDL | XXL-VLDL-L | Lipoprotein subclasses | mmol/l |
| 23426 | Total lipids in HDL | HDL-L | Total lipids | mmol/l |
| 23524 | Total lipids in IDL | IDL-L | Lipoprotein subclasses | mmol/l |
| 23559 | Total lipids in large HDL | L-HDL-L | Lipoprotein subclasses | mmol/l |
| 23531 | Total lipids in large LDL | L-LDL-L | Lipoprotein subclasses | mmol/l |
| 23496 | Total lipids in large VLDL | L-VLDL-L | Lipoprotein subclasses | mmol/l |
| 23425 | Total lipids in LDL | LDL-L | Total lipids | mmol/l |
| 23423 | Total lipids in lipoprotein particles | Total-L | Total lipids | mmol/l |
| 23566 | Total lipids in medium HDL | M-HDL-L | Lipoprotein subclasses | mmol/l |
| 23538 | Total lipids in medium LDL | M-LDL-L | Lipoprotein subclasses | mmol/l |
| 23503 | Total lipids in medium VLDL | M-VLDL-L | Lipoprotein subclasses | mmol/l |
| 23573 | Total lipids in small HDL | S-HDL-L | Lipoprotein subclasses | mmol/l |
| 23545 | Total lipids in small LDL | S-LDL-L | Lipoprotein subclasses | mmol/l |
| 23510 | Total lipids in small VLDL | S-VLDL-L | Lipoprotein subclasses | mmol/l |
| 23552 | Total lipids in very large HDL | XL-HDL-L | Lipoprotein subclasses | mmol/l |
| 23489 | Total lipids in very large VLDL | XL-VLDL-L | Lipoprotein subclasses | mmol/l |
| 23517 | Total lipids in very small VLDL | XS-VLDL-L | Lipoprotein subclasses | mmol/l |
| 23424 | Total lipids in VLDL | VLDL-L | Total lipids | mmol/l |
| 23411 | Total phospholipids in lipoprotein particles | Total-PL | Phospholipids | mmol/l |
| 23407 | Total triglycerides | Total triglycerides | Triglycerides | mmol/l |
| 23487 | Triglycerides in chylomicrons and extremely large VLDL | XXL-VLDL-TG | Lipoprotein subclasses | mmol/l |
| 23410 | Triglycerides in HDL | HDL-TG | Triglycerides | mmol/l |
| 23529 | Triglycerides in IDL | IDL-TG | Lipoprotein subclasses | mmol/l |
| 23564 | Triglycerides in large HDL | L-HDL-TG | Lipoprotein subclasses | mmol/l |
| 23536 | Triglycerides in large LDL | L-LDL-TG | Lipoprotein subclasses | mmol/l |
| 23501 | Triglycerides in large VLDL | L-VLDL-TG | Lipoprotein subclasses | mmol/l |
| 23409 | Triglycerides in LDL | LDL-TG | Triglycerides | mmol/l |
| 23571 | Triglycerides in medium HDL | M-HDL-TG | Lipoprotein subclasses | mmol/l |
| 23543 | Triglycerides in medium LDL | M-LDL-TG | Lipoprotein subclasses | mmol/l |
| 23508 | Triglycerides in medium VLDL | M-VLDL-TG | Lipoprotein subclasses | mmol/l |
| 23578 | Triglycerides in small HDL | S-HDL-TG | Lipoprotein subclasses | mmol/l |
| 23550 | Triglycerides in small LDL | S-LDL-TG | Lipoprotein subclasses | mmol/l |
| 23515 | Triglycerides in small VLDL | S-VLDL-TG | Lipoprotein subclasses | mmol/l |
| 23557 | Triglycerides in very large HDL | XL-HDL-TG | Lipoprotein subclasses | mmol/l |
| 23494 | Triglycerides in very large VLDL | XL-VLDL-TG | Lipoprotein subclasses | mmol/l |
| 23522 | Triglycerides in very small VLDL | XS-VLDL-TG | Lipoprotein subclasses | mmol/l |
| 23408 | Triglycerides in VLDL | VLDL-TG | Triglycerides | mmol/l |
| 23583 | Triglycerides to total lipids ratio in chylomicrons and extremely large VLDL | XXL-VLDL-TG % | Relative lipoprotein lipid concentrations | % |
| 23613 | Triglycerides to total lipids ratio in IDL | IDL-TG % | Relative lipoprotein lipid concentrations | % |
| 23638 | Triglycerides to total lipids ratio in large HDL | L-HDL-TG % | Relative lipoprotein lipid concentrations | % |
| 23618 | Triglycerides to total lipids ratio in large LDL | L-LDL-TG % | Relative lipoprotein lipid concentrations | % |
| 23593 | Triglycerides to total lipids ratio in large VLDL | L-VLDL-TG % | Relative lipoprotein lipid concentrations | % |
| 23643 | Triglycerides to total lipids ratio in medium HDL | M-HDL-TG % | Relative lipoprotein lipid concentrations | % |
| 23623 | Triglycerides to total lipids ratio in medium LDL | M-LDL-TG % | Relative lipoprotein lipid concentrations | % |
| 23598 | Triglycerides to total lipids ratio in medium VLDL | M-VLDL-TG % | Relative lipoprotein lipid concentrations | % |
| 23648 | Triglycerides to total lipids ratio in small HDL | S-HDL-TG % | Relative lipoprotein lipid concentrations | % |
| 23628 | Triglycerides to total lipids ratio in small LDL | S-LDL-TG % | Relative lipoprotein lipid concentrations | % |
| 23603 | Triglycerides to total lipids ratio in small VLDL | S-VLDL-TG % | Relative lipoprotein lipid concentrations | % |
| 23633 | Triglycerides to total lipids ratio in very large HDL | XL-HDL-TG % | Relative lipoprotein lipid concentrations | % |
| 23588 | Triglycerides to total lipids ratio in very large VLDL | XL-VLDL-TG % | Relative lipoprotein lipid concentrations | % |
| 23608 | Triglycerides to total lipids ratio in very small VLDL | XS-VLDL-TG % | Relative lipoprotein lipid concentrations | % |
| 23469 | Tyrosine | Tyrosine | Amino acids | mmol/l |
| 23467 | Valine | Valine | Amino acids | mmol/l |
| 23403 | VLDL cholesterol | VLDL-C | Cholesterol | mmol/l |

**Table S2.** Summary statistics and correlations of air pollutants.

| Air pollution | Mean, μg/m^3^ | SD, μg/m^3^ | Minimum, μg/m^3^ | Maximum, μg/m^3^ |
| --- | --- | --- | --- | --- |
| PM_2.5_ | 9.97 | 1.02 | 8.17 | 14.21 |
| PM_10_ | 16.21 | 1.87 | 11.78 | 23.83 |
| NO_2_ | 26.48 | 7.22 | 12.93 | 56.92 |
| NO_X_ | 43.50 | 13.95 | 19.74 | 106.24 |

Abbreviations: PM_2.5_, fine particulate; PM_10_, inhalable particulate matter; NO_2_, nitrogen dioxide; NO_x_, nitrogen oxides; SD, standard deviation.

**Table S3.** Pathway Analysis of air pollutant score-related metabolites.

| **Pathway Name** | **Total** | **-log10(P)** | **FDR** | **Impact** |
| --- | --- | --- | --- | --- |
| Glyoxylate and dicarboxylate metabolism | 4/32 | 4.0713 | 0.0067886 | 0.16333 |
| Phenylalanine, tyrosine and tryptophan biosynthesis | 2/4 | 3.4364 | 0.014646 | 1 |
| Alanine, aspartate and glutamate metabolism | 3/28 | 2.9066 | 0.026838 | 0.11378 |
| Valine, leucine and isoleucine biosynthesis | 2/8 | 2.7754 | 0.026838 | 0 |
| Phenylalanine metabolism | 2/8 | 2.7754 | 0.026838 | 0.35714 |
| Valine, leucine and isoleucine degradation | 3/40 | 2.452 | 0.04709 | 0 |

**Table S4.** Associations between pollutant mixture exposure and incident rheumatoid arthritis using quantile g-computation models.

| Analysis model | Air pollutants | | Metabolic signatures | |
| --- | --- | --- | --- | --- |
|  | HR (95% CI) | P value | HR (95% CI) | P value |
| PM_2.5_+PM_10_+NO_2_ | 1.054 (1.022,1.086) | < 0.001 | 1.188 (1.148,1.229) | < 0.001 |
| PM_2.5_+PM_10_+NO_x_ | 1.056 (1.024,1.088) | < 0.001 | 1.192 (1.152,1.233) | < 0.001 |
| PM_2.5_+PM_10_ | 1.055 (1.024,1.086) | < 0.001 | 1.187 (1.148,1.228) | < 0.001 |
| PM_2.5_+NO_2_ | 1.042 (1.014,1.071) | 0.003 | 1.205 (1.169,1.243) | < 0.001 |
| PM_2.5_+NO_x_ | 1.044 (1.017,1.073) | 0.001 | 1.216 (1.181,1.252) | < 0.001 |
| PM_10_+NO_2_ | 1.050 (1.019,1.082) | 0.001 | 1.114 (1.079,1.150) | < 0.001 |
| PM_10_+NO_x_ | 1.053 (1.022,1.084) | < 0.001 | 1.166 (1.127,1.206) | < 0.001 |

Abbreviations: PM_2.5_, fine particulate; PM_10_, inhalable particulate matter; NO_2_, nitrogen dioxide; NO_x_, nitrogen oxides

Model adjusted for age, sex, ethnicity, education levels, employment status, household income, body mass index, physical activity, smoking status, alcohol intake, healthy diet pattern, cardiovascular diseases history, diabetes history, hypertension history, estimated glomerular filtration rate, low-density lipoprotein, natural logarithm of triglycerides.

**Table S5.** The additive interactions between metabolic signature and ambient air pollution on the risk of rheumatoid arthritis.

| **Metabolic signatures** | **Additive interaction** | | | **Multiplicative interaction** |
| --- | --- | --- | --- | --- |
|  | RERI (95% CI) | AP (95% CI) | SI (95% CI) |  |
| PM_2.5_ |  |  |  |  |
| Low (< median) | - | - | - | - |
| High (≥ median) | -0.05 (-0.19, 0.09) | -0.03 (-0.13, 0.06) | 0.91 (0.70, 1.17) | 0.94 (0.83, 1.05) |
| PM_10_ |  |  |  |  |
| Low (< median) | - | - | - | - |
| High (≥ median) | 0.03 (-0.09, 0.15) | 0.02 (-0.08, 0.13) | 1.18 (0.56, 2.49) | 1.02 (0.91, 1.14) |
| NO_2_ |  |  |  |  |
| Low (< median) | - | - | - | - |
| High (≥ median) | 0.00 (-0.13, 0.14) | 0.00 (-0.09, 0.10) | 1.01 (0.69, 1.49) | 0.99 (0.88, 1.10) |
| NO_x_ |  |  |  |  |
| Low (< median) | - | - | - | - |
| High (≥ median) | -0.01(-0.15, 0.13) | -0.01 (-0.10, 0.09) | 0.98 (0.73, 1.31) | 0.97 (0.86, 1.09) |

Abbreviations: RERI, relative excess risk due to interaction. PM_2.5_, fine particulate; PM_10_, inhalable particulate matter; NO_2_, nitrogen dioxide; NO_x_, nitrogen oxides.

Model adjusted for age, sex, ethnicity, education levels, employment status, household income, body mass index, physical activity, smoking status, alcohol intake, healthy diet pattern, cardiovascular diseases history, diabetes history, hypertension history, estimated glomerular filtration rate, low-density lipoprotein, natural logarithm of triglycerides.

**Table S6.** Associations of air pollution with risk of rheumatoid arthritis using time-varying Cox regression model.

| Exposure | HR（95% CI） | *P* value |
| --- | --- | --- |
| PM_2.5_ |  |  |
| Model 1 | 1.074 (1.043-1.107) | < 0.001 |
| Model 2 | 1.064 (1.033-1.096) | < 0.001 |
| PM_10_ |  |  |
| Model 1 | 1.073 (1.046-1.102) | < 0.001 |
| Model 2 | 1.065 (1.037-1.093) | < 0.001 |
| NO_2_ |  |  |
| Model 1 | 1.062 (1.035-1.089) | < 0.001 |
| Model 2 | 1.039 (1.013-1.065) | 0.003 |
| NO_x_ |  |  |
| Model 1 | 1.056 (1.030-1.082) | < 0.001 |
| Model 2 | 1.034 (1.009-1.06) | 0.008 |

Abbreviations: PM_2.5_, particulate matter with an aerodynamic diameter ≤ 2.5 μm; PM_10_, particulate matter with an aerodynamic diameter ≤ 10 μm; NO_2_, nitrogen dioxide; NO_x_, nitrogen oxides; APS: Air pollution score; HR, hazard ratio; CI, confidence interval.

Results are presented as HRs and 95% CIs per SD increase in air pollutants.

Model 2: adjusted for age, sex, ethnicity, education levels, employment status, and household income.

Model 3: further adjusted for body mass index, physical activity, smoking status, alcohol intake, healthy diet pattern, cardiovascular diseases history, diabetes history, hypertension history, estimated glomerular filtration rate, low-density lipoprotein, natural logarithm of triglycerides.

**Table S7.** Associations of air pollution with risk of rheumatoid arthritis using Fine-gray model.

| Air pollution | SHR (95% CI) | *P* value | Metabolic signature | SHR (95% CI) | *P* value |
| --- | --- | --- | --- | --- | --- |
| PM_2.5_ | 1.04 (1.02-1.07) | 0.002 | PM_2.5_ | 1.20 (1.18-1.23) | < 0.001 |
| PM_10_ | 1.02 (1.00-1.05) | 0.083 | PM_10_ | 1.05 (1.02-1.08) | 0.001 |
| NO_2_ | 1.04 (1.01-1.07) | 0.015 | NO_2_ | 1.14 (1.11-1.17) | < 0.001 |
| NO_x_ | 1.04 (1.01-1.07) | 0.004 | NO_X_ | 1.17 (1.14-1.20) | < 0.001 |

Abbreviations: PM_2.5_, particulate matter with an aerodynamic diameter ≤ 2.5 μm; PM_10_, particulate matter with an aerodynamic diameter ≤ 10 μm; NO_2_, nitrogen dioxide; NO_x_, nitrogen oxides; APS: Air pollution score; SHR, subdistribution Hazard Ratio; CI, confidence interval.

Results are presented as SHRs and 95% CIs per SD increase in air pollutants.

Model adjusted for age, sex, ethnicity, education levels, employment status, household income, body mass index, physical activity, smoking status, alcohol intake, healthy diet pattern, cardiovascular diseases history, diabetes history, hypertension history, estimated glomerular filtration rate, low-density lipoprotein, natural logarithm of triglycerides.

**Table S8.** The associations of air pollution and related metabolic signatures with rheumatoid arthritis using E-value estimates.

|  | HR (95% CI) | *P* values | E-value |
| --- | --- | --- | --- |
| Air Pollution |  |  |  |
| PM_2.5_ | 1.046 (1.017-1.076) | 0.002 | 1.212 |
| PM_10_ | 1.025 (0.997-1.054) | 0.081 | 1.150 |
| NO_2_ | 1.039 (1.009-1.069) | 0.010 | 1.192 |
| NO_x_ | 1.043 (1.014-1.072) | 0.003 | 1.204 |
| APS | 1.045 (1.016-1.075) | 0.002 | 1.209 |
| Metabolic signature |  |  |  |
| PM_2.5_ | 1.208 (1.180-1.236) | < 0.001 | 1.539 |
| PM_10_ | 1.053 (1.021-1.086) | 0.001 | 1.231 |
| NO_2_ | 1.141 (1.109-1.173) | < 0.001 | 1.419 |
| NO_x_ | 1.174 (1.148-1.201) | < 0.001 | 1.480 |
| APS | 1.184 (1.156-1.212) | < 0.001 | 1.497 |

Abbreviations: PM_2.5_, particulate matter with an aerodynamic diameter ≤ 2.5 μm; PM_10_, particulate matter with an aerodynamic diameter ≤ 10 μm; NO_2_, nitrogen dioxide; NO_x_, nitrogen oxides; APS: Air pollution score; HR, Hazard Ratio; CI, confidence interval.

Results are presented as HRs and 95% CIs per SD increase in air pollutants.

Model adjusted for age, sex, ethnicity, education levels, employment status, household income, body mass index, physical activity, smoking status, alcohol intake, healthy diet pattern, cardiovascular diseases history, diabetes history, hypertension history, estimated glomerular filtration rate, low-density lipoprotein, natural logarithm of triglycerides.

**Table S9.** Associations of air pollutants and their related metabolic signatures with rheumatoid arthritis after multiple imputation of missing data.

| Air pollution | HR (95% CI) | *P* value | Metabolic signature | HR (95% CI) | *P* value |
| --- | --- | --- | --- | --- | --- |
| PM_2.5_ | 1.042 (1.013-1.072) | 0.004 | PM_2.5_ | 1.206 (1.179-1.235) | < 0.001 |
| PM_10_ | 1.023 (0.995-1.052) | 0.111 | PM_10_ | 1.054 (1.023-1.087) | 0.001 |
| NO_2_ | 1.036 (1.006-1.066) | 0.018 | NO_2_ | 1.173 (1.147-1.199) | < 0.001 |
| NO_x_ | 1.040 (1.011-1.069) | 0.007 | NO_X_ | 1.140 (1.108-1.172) | < 0.001 |

Abbreviations: PM_2.5_, particulate matter with an aerodynamic diameter ≤ 2.5 μm; PM_10_, particulate matter with an aerodynamic diameter ≤ 10 μm; NO_2_, nitrogen dioxide; NO_x_, nitrogen oxides; HR, Hazard Ratio; CI, confidence interval.

Results are presented as HRs and 95% CIs per SD increase in air pollutants.

Model adjusted for age, sex, ethnicity, education levels, employment status, household income, body mass index, physical activity, smoking status, alcohol intake, healthy diet pattern, cardiovascular diseases history, diabetes history, hypertension history, estimated glomerular filtration rate, low-density lipoprotein, natural logarithm of triglycerides.

**Table S10.** Association of air pollutants and related metabolic signatures with rheumatoid arthritis including fasting time as a covariate.

| Air pollution | HR (95% CI) | *P* | Metabolic signature | HR (95% CI) | *P* |
| --- | --- | --- | --- | --- | --- |
| PM_2.5_ | 1.045 (1.016-1.075) | 0.002 | PM_2.5_ | 1.207 (1.179-1.235) | < 0.001 |
| PM_10_ | 1.024 (0.996-1.053) | 0.095 | PM_10_ | 1.050 (1.018-1.083) | 0.002 |
| NO_2_ | 1.037 (1.007-1.068) | 0.014 | NO_2_ | 1.172 (1.146-1.200) | < 0.001 |
| NO_x_ | 1.042 (1.013-1.072) | 0.004 | NO_X_ | 1.138 (1.106-1.171) | < 0.001 |

Abbreviations: PM_2.5_, particulate matter with an aerodynamic diameter ≤ 2.5 μm; PM_10_, particulate matter with an aerodynamic diameter ≤ 10 μm; NO_2_, nitrogen dioxide; NO_x_, nitrogen oxides; APS: Air pollution score; HR, Hazard Ratio; CI, confidence interval.

Results are presented as HRs and 95% CIs per SD increase in air pollutants.

Model adjusted for age, sex, ethnicity, education levels, employment status, household income, body mass index, physical activity, smoking status, alcohol intake, healthy diet pattern, cardiovascular diseases history, diabetes history, hypertension history, estimated glomerular filtration rate, low-density lipoprotein, natural logarithm of triglycerides, and fasting time.

**Table S11** Associations of air pollutants and their related metabolic signatures with rheumatoid arthritis after adjusted for natural environment, green space, and water space.

|  | 300m buffer | | 1000m buffer | |
| --- | --- | --- | --- | --- |
| Air pollution | HR (95% CI) | *P* value | HR (95% CI) | *P* value |
| PM_2.5_ | 1.058 (1.019-1.099) | 0.004 | 1.073 (1.032-1.115) | < 0.001 |
| PM_10_ | 1.029 (0.997-1.062) | 0.078 | 1.030 (0.999-1.063) | 0.059 |
| NO_2_ | 1.040 (1.000-1.082) | 0.048 | 1.065 (1.017-1.114) | 0.007 |
| NO_x_ | 1.045 (1.009-1.082) | 0.015 | 1.053 (1.016-1.092) | 0.004 |
| Metabolic signature |  |  |  |  |
| PM_2.5_ | 1.211 (1.181-1.241) | < 0.001 | 1.211 (1.182-1.241) | < 0.001 |
| PM_10_ | 1.046 (1.013-1.080) | 0.006 | 1.047 (1.015-1.081) | 0.004 |
| NO_2_ | 1.177 (1.149-1.206) | < 0.001 | 1.178 (1.150-1.207) | < 0.001 |
| NO_x_ | 1.139 (1.106-1.173) | < 0.001 | 1.141 (1.108-1.175) | < 0.001 |

Abbreviations: PM_2.5_, particulate matter with an aerodynamic diameter ≤ 2.5 μm; PM_10_, particulate matter with an aerodynamic diameter ≤ 10 μm; NO_2_, nitrogen dioxide; NO_x_, nitrogen oxides; HR, Hazard Ratio; CI, confidence interval.

Results are presented as HRs and 95% CIs per SD increase in air pollutants.

Model adjusted for age, sex, ethnicity, education levels, employment status, household income, body mass index, physical activity, smoking status, alcohol intake, healthy diet pattern, cardiovascular diseases history, diabetes history, hypertension history, estimated glomerular filtration rate, low-density lipoprotein, natural logarithm of triglycerides, natural environment, green space, and water space.

**Table S12.** Associations of air pollutants and their related metabolic signatures with rheumatoid arthritis after excluding events at baseline and within the first 1 and 2 years of follow-up.

|  | 0-year lag | | 1-year lag | | 2-year lag | |
| --- | --- | --- | --- | --- | --- | --- |
| Air pollution | HR (95% CI) | *P* value | HR (95% CI) | P value | HR (95% CI) | *P* value |
| PM_2.5_ | 1.03 (1.00-1.05) | 0.018 | 1.03 (1.01-1.06) | 0.016 | 1.03 (1.00-1.06) | 0.021 |
| PM_10_ | 1.02 (0.99-1.04) | 0.161 | 1.02 (0.99-1.04) | 0.229 | 1.02 (0.99-1.04) | 0.195 |
| NO_2_ | 1.03 (1.00-1.05) | 0.029 | 1.03 (1.00-1.06) | 0.023 | 1.03 (1.00-1.06) | 0.024 |
| NO_x_ | 1.03 (1.01-1.06) | 0.007 | 1.03 (1.01-1.06) | 0.007 | 1.04 (1.01-1.06) | 0.007 |
| Metabolic signature |  |  |  |  |  |  |
| PM_2.5_ | 1.23 (1.21-1.26) | < 0.001 | 1.23 (1.20-1.25) | < 0.001 | 1.22 (1.20-1.25) | < 0.001 |
| PM_10_ | 1.07 (1.04-1.09) | < 0.001 | 1.06 (1.03-1.09) | < 0.001 | 1.06 (1.03-1.09) | < 0.001 |
| NO_2_ | 1.16 (1.14-1.19) | < 0.001 | 1.16 (1.13-1.18) | < 0.001 | 1.15 (1.12-1.18) | < 0.001 |
| NO_x_ | 1.19 (1.17-1.22) | < 0.001 | 1.19 (1.17-1.21) | < 0.001 | 1.19 (1.16-1.21) | < 0.001 |

Abbreviations: PM_2.5_, particulate matter with an aerodynamic diameter ≤ 2.5 μm; PM_10_, particulate matter with an aerodynamic diameter ≤ 10 μm; NO_2_, nitrogen dioxide; NO_x_, nitrogen oxides; HR, Hazard Ratio; CI, confidence interval.

Results are presented as HRs and 95% CIs per SD increase in air pollutants.

Model adjusted for age, sex, ethnicity, education levels, employment status, household income, body mass index, physical activity, smoking status, alcohol intake, healthy diet pattern, cardiovascular diseases history, diabetes history, hypertension history, estimated glomerular filtration rate, low-density lipoprotein, natural logarithm of triglycerides.

**Table S13.** Association of air pollutants with rheumatoid arthritis using the 1-year, 2-year and 3-year exposure window.

|  | 1-year exposure windows | | 2-year exposure windows | | 3-year exposure windows | |
| --- | --- | --- | --- | --- | --- | --- |
|  | HR (95% CI) | *P* value | HR (95% CI) | *P* value | HR (95% CI) | *P* value |
| PM_2.5_ | 1.069 (1.045-1.093) | < 0.001 | 1.048 (1.024-1.072) | < 0.001 | 1.050 (1.027-1.075) | < 0.001 |
| PM_10_ | 1.051 (1.027-1.075) | < 0.001 | 1.042 (1.018-1.066) | < 0.001 | 1.045 (1.021-1.069) | < 0.001 |
| NO_2_ | 1.035 (1.012-1.059) | 0.003 | 1.036 (1.013-1.060) | 0.002 | 1.031 (1.007-1.056) | 0.013 |
| NO_x_ | 1.027 (1.004-1.050) | 0.023 | 1.032 (1.009-1.056) | 0.006 | 1.027 (1.003-1.052) | 0.026 |

Abbreviations: PM_2.5_, particulate matter with an aerodynamic diameter ≤ 2.5 μm; PM_10_, particulate matter with an aerodynamic diameter ≤ 10 μm; NO_2_, nitrogen dioxide; NO_x_, nitrogen oxides; HR, Hazard Ratio; CI, confidence interval.

Results are presented as HRs and 95% CIs per SD increase in air pollutants.

Model adjusted for age, sex, ethnicity, education levels, employment status, household income, body mass index, physical activity, smoking status, alcohol intake, healthy diet pattern, cardiovascular diseases history, diabetes history, hypertension history, estimated glomerular filtration rate, low-density lipoprotein, natural logarithm of triglycerides.

**Table S14.** Associations of air pollutants and their related metabolic signatures with rheumatoid arthritis after excluding LDL cholesterol and triglycerides from the covariate set.

| Air pollution | HR (95% CI) | *P* | Metabolic signature | HR (95% CI) | *P* |
| --- | --- | --- | --- | --- | --- |
| PM_2.5_ | 1.047 (1.018-1.077) | 0.001 | PM_2.5_ | 1.208 (1.181-1.236) | < 0.001 |
| PM_10_ | 1.026 (0.998-1.055) | 0.073 | PM_10_ | 1.065 (1.035-1.096) | < 0.001 |
| NO_2_ | 1.040 (1.010-1.070) | 0.008 | NO_2_ | 1.172 (1.146-1.198) | < 0.001 |
| NO_x_ | 1.044 (1.015-1.074) | 0.003 | NO_X_ | 1.135 (1.105-1.166) | < 0.001 |

Abbreviations: PM_2.5_, particulate matter with an aerodynamic diameter ≤ 2.5 μm; PM_10_, particulate matter with an aerodynamic diameter ≤ 10 μm; NO_2_, nitrogen dioxide; NO_x_, nitrogen oxides; APS: Air pollution score; HR, Hazard Ratio; CI, confidence interval.

Results are presented as HRs and 95% CIs per SD increase in air pollutants.

Model adjusted for age, sex, ethnicity, education levels, employment status, household income, body mass index, physical activity, smoking status, alcohol intake, healthy diet pattern, cardiovascular diseases history, diabetes history, hypertension history, and estimated glomerular filtration rate.

**Table S15.** Associations between equal-weighted air pollution score and rheumatoid arthritis risk.

| Air pollution score | HR (95% CI) | *P* |
| --- | --- | --- |
| Tertile 1 | Ref |  |
| Tertile 2 | 1.077 (1.005-1.155) | 0.036 |
| Tertile 3 | 1.111 (1.035-1.192) | 0.003 |
| P for trend |  | 0.004 |
| Per SD increment | 1.045 (1.015-1.075) | 0.003 |

Abbreviations: HR, Hazard Ratio; CI, confidence interval.

Results are presented as HRs and 95% CIs per SD increase in air pollutants.

Model adjusted for age, sex, ethnicity, education levels, employment status, household income, body mass index, physical activity, smoking status, alcohol intake, healthy diet pattern, cardiovascular diseases history, diabetes history, hypertension history, estimated glomerular filtration rate, low-density lipoprotein, natural logarithm of triglycerides.

**Figure S1.** Flowchart of participant selection for the study

**Figure S2.** Direct acyclic graph of the association between air pollution and rheumatoid arthritis risk.

**Figure S3.** Associations of metabolites comprising the PM_2.5_-related metabolic signature with rheumatoid arthritis and air pollutants.

Abbreviations: PM_2.5_, particulate matter with an aerodynamic diameter ≤ 2.5 μm; PM_10_, particulate matter with an aerodynamic diameter ≤ 10 μm; NO_2_, nitrogen dioxide; NO_x_, nitrogen oxides; APS, air pollutant score. A. metabolite coefficients from LASSO regression in the PM_2.5_-related metabolic signature. B. The effects of metabolites comprising the PM_2.5_-related metabolic signature on rheumatoid arthritis using Cox regression model. Model adjusted for age, sex, ethnicity, education levels, employment status, household income, body mass index, physical activity, smoking status, alcohol intake, healthy diet pattern, cardiovascular diseases history, diabetes history, hypertension history, estimated glomerular filtration rate, low-density lipoprotein, natural logarithm of triglycerides. The effect size is presented as log(HR), with 95% confidence intervals shown as error bars. Red bars indicate an increased risk, whereas blue bars indicate a decreased risk. Statistical significance is denoted by asterisks (^*^*P* < 0.05, ^**^*P* < 0.01, ^***^*P* < 0.001). C. The effects of air pollutant and PM_2.5_ on metabolic signatures using linear regression model. The model adjustments are consistent with model in Plot B. The effect size is presented as beta and the significance is labeled by asterisks (^*^*P* < 0.05, ^**^*P* < 0.01, ^***^*P* < 0.001).

**Figure S4.** Associations of metabolites comprising the PM_10_-related metabolic signature with rheumatoid arthritis and air pollutants.

Abbreviations: PM_2.5_, particulate matter with an aerodynamic diameter ≤ 2.5 μm; PM_10_, particulate matter with an aerodynamic diameter ≤ 10 μm; NO_2_, nitrogen dioxide; NO_x_, nitrogen oxides. A. Metabolite coefficients from LASO regression in the PM_10_-related metabolic signature. B. The effects of metabolites comprising the PM_10_-related metabolic signature on rheumatoid arthritis using Cox regression model. Model adjusted for age, sex, ethnicity, education levels, employment status, household income, body mass index, physical activity, smoking status, alcohol intake, healthy diet pattern, cardiovascular diseases history, diabetes history, hypertension history, estimated glomerular filtration rate, low-density lipoprotein, natural logarithm of triglycerides. The effect size is presented as log(HR), with 95% confidence intervals shown as error bars. Red bars indicate an increased risk, whereas blue bars indicate a decreased risk. Statistical significance is denoted by asterisks (^*^*P* < 0.05, ^**^*P* < 0.01, ^***^*P* < 0.001). C. The effects of air pollutant and PM_10_ on metabolic signatures using linear regression model. The model adjustments are consistent with model in Plot B. The effect size is presented as beta and the significance is labeled by asterisks (^*^*P* < 0.05, ^**^*P* < 0.01, ^***^*P* < 0.001).

**Figure S5.** Associations of metabolites comprising the NO_2_-related metabolic signature with rheumatoid arthritis and air pollutants.

Abbreviations: PM_2.5_, particulate matter with an aerodynamic diameter ≤ 2.5 μm; PM_10_, particulate matter with an aerodynamic diameter ≤ 10 μm; NO_2_, nitrogen dioxide; NO_x_, nitrogen oxides. A. Metabolite coefficients from LASSO regression in the PM_10_-related metabolic signature. B. The effects of metabolites comprising the NO_2_-related metabolic signature on rheumatoid arthritis using Cox regression model. Model adjusted for age, sex, ethnicity, education levels, employment status, household income, body mass index, physical activity, smoking status, alcohol intake, healthy diet pattern, cardiovascular diseases history, diabetes history, hypertension history, estimated glomerular filtration rate, low-density lipoprotein, natural logarithm of triglycerides. The effect size is presented as log(HR), with 95% confidence intervals shown as error bars. Red bars indicate an increased risk, whereas blue bars indicate a decreased risk. Statistical significance is denoted by asterisks (^*^*P* < 0.05, ^**^*P* < 0.01, ^***^*P* < 0.001). C. The effects of air pollutant and NO_2_ on metabolic signatures using linear regression model. The model adjustments are consistent with model in Plot B. The effect size is presented as beta and the significance is labeled by asterisks (^*^*P* < 0.05, ^**^*P* < 0.01, ^***^*P* < 0.001).

**Figure S6.** Associations of metabolites comprising the NO_x_-related metabolic signature with rheumatoid arthritis and air pollutants.

Abbreviations: PM_2.5_, particulate matter with an aerodynamic diameter ≤ 2.5 μm; PM_10_, particulate matter with an aerodynamic diameter ≤ 10 μm; NO_2_, nitrogen dioxide; NO_x_, nitrogen oxides. A. Metabolite coefficients from LASSO regression in the PM_10_-related metabolic signature. B. The effects of metabolites comprising the NO_x_-related metabolic signature on rheumatoid arthritis using Cox regression model. Model adjusted for age, sex, ethnicity, education levels, employment status, household income, body mass index, physical activity, smoking status, alcohol intake, healthy diet pattern, cardiovascular diseases history, diabetes history, hypertension history, estimated glomerular filtration rate, low-density lipoprotein, natural logarithm of triglycerides. The effect size is presented as log(HR), with 95% confidence intervals shown as error bars. Red bars indicate an increased risk, whereas blue bars indicate a decreased risk. Statistical significance is denoted by asterisks (^*^*P* < 0.05, ^**^*P* < 0.01, ^***^*P* < 0.001). C. The effects of air pollutant and NO_x_ on metabolic signatures using linear regression model. The model adjustments are consistent with model in Plot B. The effect size is presented as beta and the significance is labeled by asterisks (^*^*P* < 0.05, ^**^*P* < 0.01, ^***^*P* < 0.001).

**Figure S7.** The composition of air pollution-related metabolic signatures.

Abbreviations: PM_2.5_, particulate matter with an aerodynamic diameter ≤ 2.5 μm; PM_10_, particulate matter with an aerodynamic diameter ≤ 10 μm; NO_2_, nitrogen dioxide; NO_x_, nitrogen oxides.

**Figure S8.** Subgroup analyses for the association of air pollutants with rheumatoid arthritis

Abbreviations: PM_2.5_, particulate matter with an aerodynamic diameter ≤ 2.5 μm; PM_10_, particulate matter with an aerodynamic diameter ≤ 10 μm; NO_2_, nitrogen dioxide; NO_x_, nitrogen oxides; HR, Hazard Ratio; CI, confidence interval.

Results are presented as HRs and 95% CIs per SD increase in air pollutants.

Model adjusted for age, sex, ethnicity, education levels, employment status, household income, body mass index, physical activity, smoking status, alcohol intake, healthy diet pattern, cardiovascular diseases history, diabetes history, hypertension history, estimated glomerular filtration rate, low-density lipoprotein, natural logarithm of triglycerides (excluding subgroup variable).

**Figure S9.** Exposure–response relationships of air pollution and related metabolic signatures with rheumatoid arthritis risk based on restricted cubic spline models with knots at the 10th, 50th, and 90th percentiles.

Abbreviations: PM_2.5_, particulate matter with an aerodynamic diameter ≤ 2.5 μm; PM_10_, particulate matter with an aerodynamic diameter ≤ 10 μm; NO_2_, nitrogen dioxide; NO_x_, nitrogen oxides. Model adjusted for age, sex, ethnicity, education levels, employment status, household income, body mass index, physical activity, smoking status, alcohol intake, healthy diet pattern, cardiovascular diseases history, diabetes history, hypertension history, estimated glomerular filtration rate, low-density lipoprotein, natural logarithm of natural logarithm of triglycerides.

**Supplementary Methods**

**1. Metabolite preprocessing**

All metabolic biomarkers were standardized to Z scores within the analytic sample before statistical modeling to ensure comparability across biomarkers measured on different scales. For metabolites with missing values, concentrations were considered to be below the detection limit and were replaced by half of the minimum detectable value, consistent with previous studies using the UK Biobank NMR metabolomics platform.

**2. Construction of air pollution-related metabolic signatures**

To identify metabolomic profiles associated with ambient air pollution, least absolute shrinkage and selection operator (LASSO) regression was performed using the glmnet package in R. For each air pollutant, including PM_2.5_, PM_10_, NO_2_, NO_x_, and the air pollution score (APS), the pollutant concentration or APS was modeled as the dependent variable, and standardized metabolic biomarkers were entered as candidate predictors.

The tuning parameter λ was selected using 10-fold cross-validation. Specifically, we used λ1se, defined as the largest value of λ within one standard error of the minimum cross-validated error, rather than λmin. This criterion was selected to obtain a more parsimonious and stable set of metabolites and to reduce potential overfitting. Metabolites with non-zero coefficients at λ1se were retained as pollutant-related metabolites.

For each pollutant, the corresponding metabolic signature was calculated as a weighted linear combination of the selected metabolites:

Metabolic signature = $\sum(\beta_{i}\times{metabolite}_{i})$

where β_i_ represents the LASSO-derived coefficient of the i_th_ selected metabolite. The resulting metabolic signatures were standardized to Z scores before subsequent association analyses.

**3. Association analyses**

Cox proportional hazards regression models were used to estimate the associations of air pollutants and pollutant-related metabolic signatures with incident rheumatoid arthritis (RA). Follow-up time was calculated from baseline assessment to the first diagnosis of RA, death, loss to follow-up, or the end of follow-up, whichever occurred first. Effect estimates were presented as hazard ratios (HRs) and 95% confidence intervals (CIs).

Three sequential models were constructed. Model 1 was adjusted for age, sex, and ethnicity. Model 2 was further adjusted for educational attainment, employment status, and household income. Model 3 was additionally adjusted for body mass index, physical activity, smoking status, alcohol intake, healthy dietary pattern, low-density lipoprotein cholesterol, natural log-transformed triglycerides, diabetes, hypertension, cardiovascular disease, and estimated glomerular filtration rate. The proportional hazards assumption was assessed using Schoenfeld residuals.

Associations between air pollutants and individual metabolites were examined using multivariable linear regression models with the same covariate adjustment strategy as described above. Associations between selected metabolites and incident RA were assessed using Cox proportional hazards models. To account for multiple testing in metabolite-level analyses, false discovery rate correction was applied where appropriate.

**4. Restricted cubic spline analysis**

Potential non-linear exposure–response relationships between air pollutants and RA risk were evaluated using restricted cubic spline models. Three knots were placed at the 10_th_, 50_th_, and 90_th_ percentiles of the exposure distribution[1,2]. Tests for overall and non-linear associations were performed by comparing models with and without spline terms.

**5. Mixture analysis**

Quantile-based g-computation was used to estimate the joint associations of multiple air pollutants and metabolic signature mixtures with incident RA. This approach estimates the overall association associated with a simultaneous one-quantile increase in all components of the mixture while allowing individual components to have effects in different directions. Mixture analyses were performed for air pollutant combinations and for pollutant-related metabolic signature combinations.

**6. Generalized propensity score weighting**

To improve covariate balance for measured confounders in analyses of continuous air pollution exposures and metabolic signatures, generalized propensity score weighting was applied. Balancing weights were estimated using generalized linear models including the main covariates [3,4]. Extreme weights were truncated at the 5th and 95th percentiles to improve model stability. Weighted Cox regression models with robust standard errors were then used to estimate associations with incident RA.

**7. Mediation analysis**

Exploratory mediation analyses were conducted to evaluate whether baseline metabolic signatures or individual metabolites partly explained the associations between long-term air pollution exposure and incident RA. These analyses were based on the assumed temporal sequence in which long-term residential air pollution exposure may influence baseline circulating metabolic profiles, which may subsequently be associated with future RA risk during follow-up.

For each mediation analysis, the exposure–mediator association was estimated using linear regression, and the mediator–outcome association was estimated using Cox proportional hazards regression. The same covariate set was included in both models. The indirect effect, direct effect, total effect, and proportion mediated were estimated. Given that metabolomic profiles were measured only once at baseline and that the temporal ordering between exposure and metabolic alterations could not be fully established, mediation results were interpreted as exploratory evidence of potential biological pathways rather than definitive causal mediation.

**8. Interaction analysis**

The joint influence of air pollution exposure and pollutant-related metabolic signatures on RA risk was evaluated on both additive and multiplicative scales. Additive interaction was assessed using the relative excess risk due to interaction (RERI). Multiplicative interaction was evaluated by including product terms in Cox models and comparing models with and without interaction terms using likelihood ratio tests.

**9. Pathway analysis**

Metabolic biomarkers showing significant mediation effects were entered into pathway analysis using MetaboAnalyst 6.0. Pathway annotation was based on KEGG, HMDB, and PubChem. Pathways with false discovery rate-adjusted P values below 0.05 were considered statistically significant.

**10. Sensitivity analyses**

Several sensitivity analyses were conducted to evaluate the robustness of the findings. First, annual air pollutant concentrations were incorporated as time-updated exposures in time-varying Cox models. Second, Fine–Gray subdistribution hazard models were used to account for all-cause mortality as a competing event. Third, E-values were calculated to assess the potential impact of unmeasured confounding. Fourth, fasting duration was additionally adjusted for in the models. Fifth, alternative lag-period analyses were performed by excluding participants with prevalent RA at baseline and those who developed RA within the first 1 or 2 years of follow-up. Sixth, missing covariate values were addressed using multiple imputation by chained equations. Seventh, additional adjustment was made for natural environment, green space, and water space. Eighth, we refitted the main models without LDL cholesterol and natural log-transformed triglycerides to assess whether adjustment for lipid parameters influenced the associations between air pollution-related metabolic signatures and incident RA. Finally, alternative exposure windows based on 1-year, 2-year, and 3-year average air pollutant concentrations were examined. Eighth, an equal-weighted APS was calculated as the average of standardized PM_2.5_, NO_2_, and NO_x_ concentrations.

All statistical analyses were performed using R version 4.4.1. Two-sided *P* values <0.05 were considered statistically significant.

**Reference:**

[1] Shi, T., Yang, C., Fan, Z., et al., 2026. Circulating cardiometabolic metabolite profiles associated with ambient air pollution and atrial fibrillation risk: a prospective cohort study, Cardiovascular diabetology.

[2] Hu, P., Wei, S., Chen, G., et al., 2025. Mapping plasma metabolic profiles reflecting air pollution and their association with kidney failure: A prospective cohort study, Journal of hazardous materials, 500, 140506.

[3] Austin, P.C., 2011. An Introduction to Propensity Score Methods for Reducing the Effects of Confounding in Observational Studies, Multivariate behavioral research, 46, 399-424.

[4] Austin, P.C., Stuart, E.A., 2015. Moving towards best practice when using inverse probability of treatment weighting (IPTW) using the propensity score to estimate causal treatment effects in observational studies, Statistics in Medicine, 34, 3661-3679.
